# Abundance and co-occurrence of extracellular capsules increase environmental breadth: Implications for the emergence of pathogens

**DOI:** 10.1371/journal.ppat.1006525

**Published:** 2017-07-24

**Authors:** Olaya Rendueles, Marc Garcia-Garcerà, Bertrand Néron, Marie Touchon, Eduardo P. C. Rocha

**Affiliations:** 1 Microbial Evolutionary Genomics, Institut Pasteur, Paris, France; 2 UMR 3525, CNRS, Paris, France; 3 C3Bi, CIB, Institut Pasteur, Paris, France; New York Medical College, UNITED STATES

## Abstract

Extracellular capsules constitute the outermost layer of many bacteria, are major virulence factors, and affect antimicrobial therapies. They have been used as epidemiological markers and recently became vaccination targets. Despite the efforts to biochemically serotype capsules in a few model pathogens, little is known of their taxonomic and environmental distribution. We developed, validated, and made available a computational tool, CapsuleFinder, to identify capsules in genomes. The analysis of over 2500 prokaryotic genomes, accessible in a database, revealed that *ca*. 50% of them—including Archaea—encode a capsule. The Wzx/Wzy-dependent capsular group was by far the most abundant. Surprisingly, a fifth of the genomes encode more than one capsule system—often from different groups—and their non-random co-occurrence suggests the existence of negative and positive epistatic interactions. To understand the role of multiple capsules, we queried more than 6700 metagenomes for the presence of species encoding capsules and showed that their distribution varied between environmental categories and, within the human microbiome, between body locations. Species encoding capsules, and especially those encoding multiple capsules, had larger environmental breadths than the other species. Accordingly, capsules were more frequent in environmental bacteria than in pathogens and, within the latter, they were more frequent among facultative pathogens. Nevertheless, capsules were frequent in clinical samples, and were usually associated with fast-growing bacteria with high infectious doses. Our results suggest that capsules increase the environmental range of bacteria and make them more resilient to environmental perturbations. Capsules might allow opportunistic pathogens to profit from empty ecological niches or environmental perturbations, such as those resulting from antibiotic therapy, to colonize the host. Capsule-associated virulence might thus be a by-product of environmental adaptation. Understanding the role of capsules in natural environments might enlighten their function in pathogenesis.

## Author summary

Extracellular capsules protect bacterial cells from external aggressions such as antibiotics or desiccation, but can also be targeted by vaccines. Since little was known about their frequency across Prokaryotes, we created and made freely available a computational tool, CapsuleFinder, to identify them from genomic data. Surprisingly, its use showed that many bacterial strains, especially those with the largest genomes, encode several capsules. The frequencies of the different combinations of capsule groups depended strongly on the phyla and the groups themselves, suggesting the existence of epistatic interactions between capsules. Bacteria encoding capsule systems were found in many natural environments, and were frequent in the human microbiome. In contrast to their frequent association with virulence, we found many more capsules in non-pathogens or facultative pathogens than among obligatory pathogens. We suggest that capsules increase the environmental breadth of bacteria thereby facilitating host colonization by opportunistic pathogens.

## Introduction

Extracellular capsules, hereafter named capsules, constitute the outermost layer of some prokaryotic cells where they establish the first contact between the microorganism and its environment. They fullfill a myriad of roles, often linked to colonization and persistence. Their physical properties prevent dessication by retaining moisture near the cell surface, enhance survival in harsh environments, and protect cells from phagocytosis by grazing protozoa [1–4]. Capsules also play an essential role during infection; they downregulate pro-inflammatory cytokines [5], protect cells against reactive oxygen species generated by the host [6], and help bacteria to evade phagocytosis by macrophages and complement activation [3]. Capsules also reduce the efficiency of antibiotics [7] and cationic antimicrobial peptides [8]. These medical implications have driven the research on capsules and their roles, leading to the widespread perception that they are mostly associated with virulence [9, 10]. This triggered the numerous studies on the genetic diversity of capsules in several prominent bacterial pathogens such as *Streptococcus pneumoniae* [11, 12], *Escherichia coli* [13], *Klebsiella pneumoniae* [14, 15], *Campylobacter jejuni* [16], and *Acinetobacter baumanii* [17].

**Fig 1:**
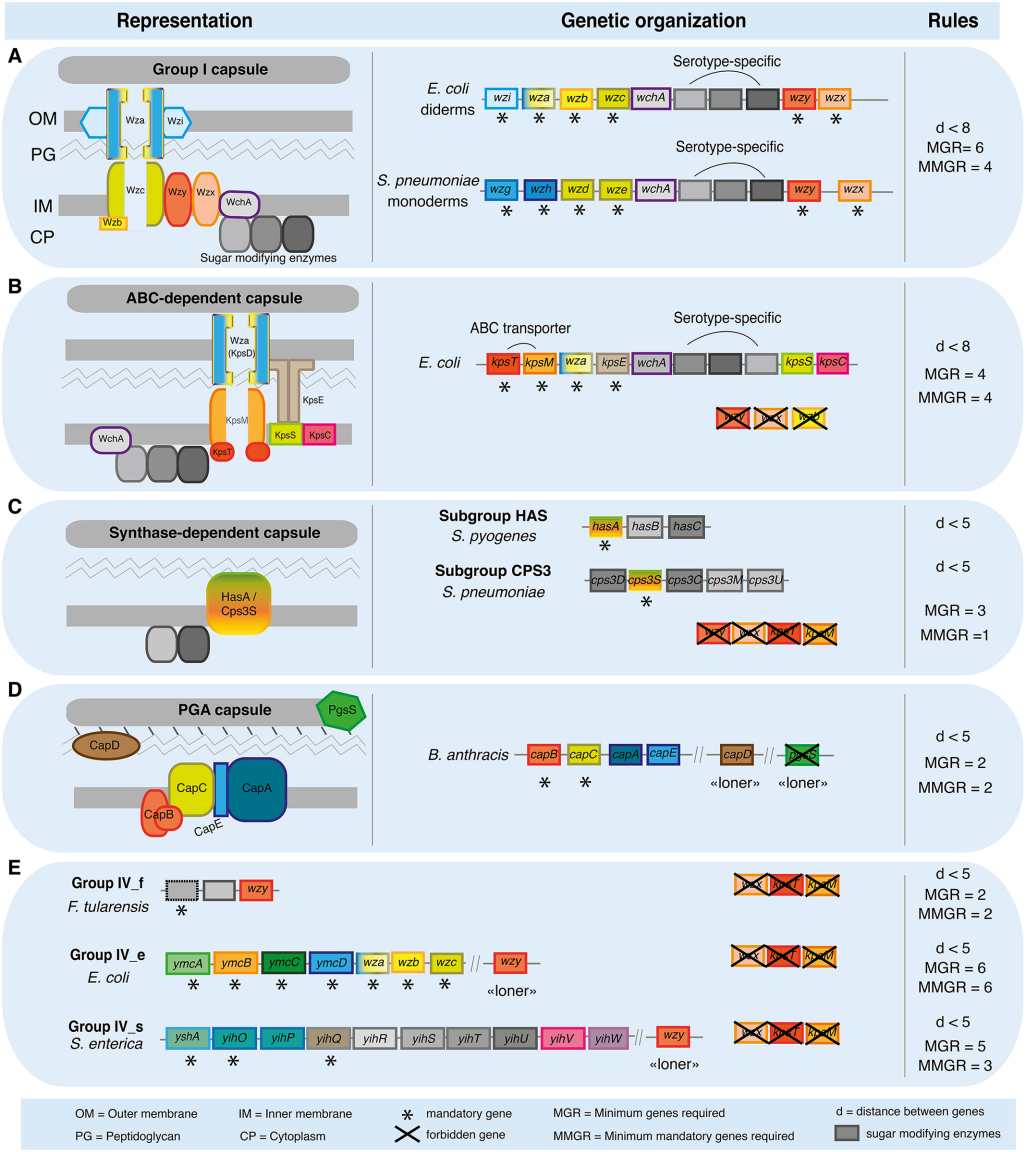
Schema of the components of capsule biosynthesis, genetic organization of the corresponding genes, and model rules for Group I (A), ABC-dependent (groups II and III) (B), synthase-dependent (C), PGA (D) and Group IV (E) capsules. **The schema** describes the putative position of the components in relation to the cell envelope. **The genetic organization** describes the genes for each component and their class ("mandatory", "accessory", "forbidden"). The crosses on the boxes indicate "forbidden components", *i*.*e*., components whose presence in the system indicate that this is not a capsule of the corresponding group. The attribute "loner" indicates that the component can be encoded elsewhere in the genome. The **model rules** indicate the maximal distance between components of the locus (d), and the minimum quorum of mandatory and accessory genes.

Capsules can be synthesized through different genetic pathways (Fig 1 and reviewed in [18–20]). Most capsules are high molecular weight polysaccharides made up of repeat units of oligosaccharides. In capsules synthesized through the Wzx/Wzy-dependent pathway or Group I [20], the oligosaccharidic repeat unit is linked to an undecaprenyl phosphate acceptor in the cytoplasm by membrane-bound glycosyltransferases. This precursor is then transported across the inner membrane by the Wzx flippase and polymerized nonprocessively in the periplasm by the Wzy polymerase. In contrast, the nascent polysaccharidic chains of Group II and Group III capsules are polymerized in the cytoplasm and linked to a phospholipid acceptor before being transported across the inner membrane by the ATP-binding cassette (ABC) transporter. Group II and III capsules will be jointly referred to as ABC-dependent capsules. In spite of these differences, both the Wzx/Wzy- and the ABC- dependent pathways use homologous outer membrane proteins from the polysaccharide export family to transport the capsule across the outer membrane of diderm bacteria [21]. Both pathways are characterized by large operons that have a conserved region encoding the secretion machinery and a variable region encoding numerous polymer-specific enzymes. The latter defines the capsule serotype and includes enzymes for the synthesis of NDP-sugars, glycosidic linkages (mainly by glycosyl-transferases), and sugar modification (O-acetylation). Within-species serotype-diversity prompted the biochemical characterization of the oligosaccharide composition of capsules, ultimately leading to the development of serotype-specific vaccines [16, 22, 23], and serotyping schemes for epidemic strains [24, 25]. The synthesis of the polysaccharidic Group IV capsules relies on the Wzy polymerase but not on Wzx flippase, and depends on very diverse export machineries, including in certain cases proteins homologous to those of Group I [26, 27]. Polysaccharidic capsules can also be produced by the synthase-dependent pathway, where a unique processive enzyme is responsible for the all the steps of initiation, polymerization and translocation of the capsule [28]. Some capsules are proteic, instead of polysaccharidic, notably the poly-γ-d-glutamate or PGA capsules produced by *Bacillus anthracis* [29].

To date very few studies have characterized the frequency and diversity of capsules across bacterial phyla, presumably because they are difficult to identify. Capsular systems have many poorly characterized components and are subject to frequent variation by homologous recombination and horizontal transfer, resulting in rapid genetic turnover [30]. Furthermore, the genetic pathways leading to the synthesis of lipopolysaccharides (LPS), extracellular polysaccharides (EPS), and capsules have many key homologous components that are difficult to disentangle [31, 32]. Finally, there are few studies on the role of capsules in ecological settings other than the host, limiting the identification of new capsule secretion pathways.

The understanding of capsule distribution and evolution across Prokaryotes has been hampered by the lack of computational tools to identify capsule systems in genomes. In order to tackle this limitation, we have built protein profiles to identify the key components of the different capsule biosynthesis pathways and defined models describing their expected frequency and genetic organization. We used them within MacSyFinder, a computational tool that allows the detection of macromolecular systems [33], to identify capsule systems in more than 2500 complete prokaryotic genomes. We then searched for the presence of species with capsules in more than 6700 metagenomes. We aimed at answering the following questions: How many capsules are there in prokaryotic genomes? Do multiple capsule groups co-occur and, if so, are there any correlations between capsule groups? Which Prokaryotes encode capsules? Which are the genetic and life-history traits associated to capsule prevalence? What is the environmental distribution of Prokaryotes encoding capsules? Our results uncovered novel intriguing patterns in the distribution of capsules, which have important biological implications and provide new insights into the evolutionary and ecological role of capsules.

## Results

### CapsuleFinder: A tool to mine genomes for extracellular capsules

We defined independent and customizable models describing the genetic composition and organization of eight groups and subgroups of capsules (Fig 1), based on the literature of the best-described experimental capsule systems [18–21, 26, 27, 29, 34]. This information was complemented with exploratory analyses of the diversity of these systems in other genomes (see Methods). We identified 58 key components (protein families) involved in capsule synthesis. The majority of them regard the secretory and polymerization components of each capsule system, as well as the most common polymer-specific enzymes. Each component was associated with a hidden Markov model (HMM) protein profile, retrieved from PFAM (31) or built for the purpose of this study (27) (S1 Table). The resulting computational tool—CapsuleFinder—uses as input the protein sequences of a genome, searches for the components of capsule systems using the HMM profiles and then delimits the systems based on the information provided in the models.

There is no curated database with information on the organisms encoding and/or lacking capsule systems. The literature rarely mentions the absence (or presence) of a capsule for non-pathogens. Nevertheless, we sought to validate CapsuleFinder by comparing its results with those mentioned in two lists of some of the best-studied encapsulated Prokaryotes [19, 35]. We successfully identified capsules in all 11 species that were reported as encapsulated and for which a complete genome sequence was available. To validate a broader set of systems, we randomly picked 100 species from our complete genome database. We then checked the literature for information on the presence of a capsule in the 40 species where a capsule system was detected (S2 Table). There were 28 species for which we could find published reference to the presence or absence of a capsule. Among these, we found published experimental evidence for a capsule in 15 species and some positive information (from either bioinformatic analyses or evidence in closely-related species) for 10 others. The literature explicitly mentioned that no capsule had been observed for the remaining three species (details S2 Table). It is difficult to say if these are false positives, which would give a false positive rate of ~8%, or if capsules actually exist in the species and the respective strains or conditions of expression were not yet identified. We have not attempted to quantify the rate of false negatives—cases where we missed an existing capsule—since there have been very few experimental efforts to show that a species lacks a capsule in a variety of environmental conditions. Yet, the analysis of our data showed a small number of cases where we missed some capsule systems and obtained some false positives. These are indicated in S3 Table. Even in the worst case, CapsuleFinder is able to identify all the best-known capsules whilst fetching few putative false positives (S1 Text).

### Abundance and phylogenetic distribution of capsule systems

**Fig 2:**
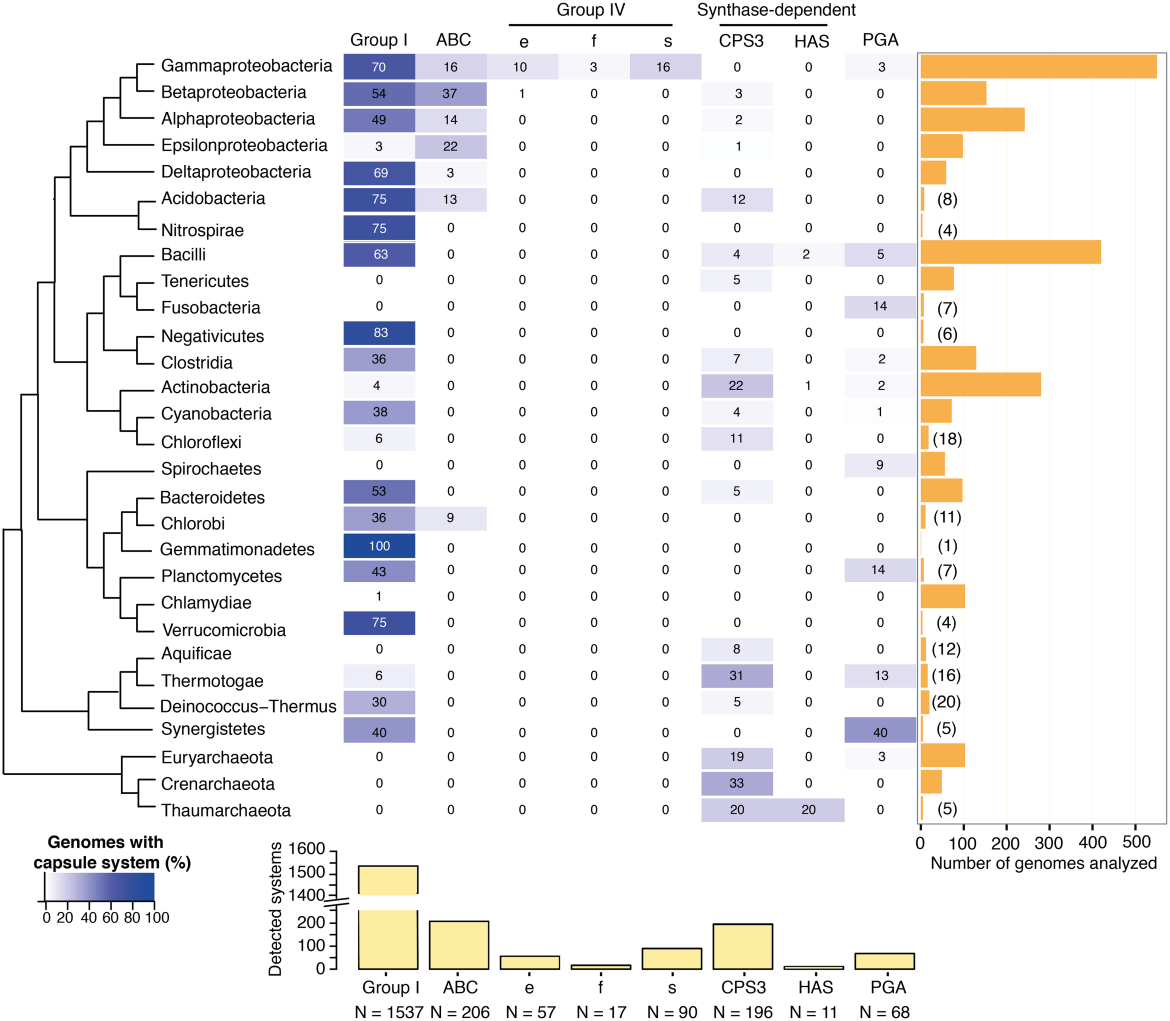
Quantification and distribution of capsule systems in Prokaryotes. The boxes indicate the percentage of genomes in the clade harbouring each system. The colours of the boxes follow a gradient from white (0%) to blue (100%) as shown in the legend. The bottom bar plot shows the total number of detected systems. The bar plot on the right represents the number of genomes per clade and in parenthesis we show the genomes that add to twenty or less. In the absence of a consensual phylogenetic tree of Prokaryotes, the cladogram was adapted from Abby et al [36] and completed with information given by [37].

We detected 2182 capsule systems in 1304 out of the 2643 genomes (Fig 2). The complete list of genomes and capsule systems is available in S1 Dataset. Around half (49%) of the genomes, representing 52% of the species, encoded at least one capsule system. Group I capsules were the most frequent, representing *ca*. 70% of the total. ABC-dependent and synthase-dependent capsules were less frequent (nearly 10% each), and subgroup CPS3 capsules were the most frequent among the latter. Group IV capsules (8.8%), and PGA proteic capsules were rarer (3.1%) (Fig 2 and S1 Dataset).

We investigated the presence of capsule systems in all major taxonomic divisions of Bacteria and Archaea (Fig 2). The highly abundant Group I capsule was detected in all bacterial phyla represented by more than 20 available complete genomes (except *Spirochaetes* and *Tenericutes*). PGA capsules, even if rare overall, were also present in most phyla. They were particularly abundant in Synergistetes, Planctomycetes, Bacillales and Fusobacteria (Fig 2). On the other hand, Group IV capsules were almost exclusively identified in γ-Proteobacteria and some subgroups were only identified in the taxa in which they were first described, *e*.*g*., all Group IV_f capsules were identified in *Francisella* spp. (Fig 2). We identified at least five out of the eight capsule groups in α- and γ-Proteobacteria and in Actinobacteria. Following previous observations of capsule-like structures in Archaea [38–40], and even if no experimental evidence has yet been given for their existence, we detected 47 capsule systems in 40 archaeal genomes. They were all synthase-dependent (both subgroups) or PGA capsules. Taken together, our results show that capsules are prevalent in Prokaryotes, where their frequency depends on the capsule group and on the phyla.

### Capsule complexity does not correlate with genome size

The genetic loci encoding the experimentally studied capsule systems have remarkably different sizes. Since the number of genes in the capsule system is expected to have some impact on the complexity and evolution of capsules, we computed the number of genes of each system identified in our work (see Materials and methods). These values are only approximate, because capsule systems surrounded by genes encoding enzymes involved in sugar metabolism cannot be delimited without ambiguity in the absence of experimental work. The Group I and ABC-dependent capsules were encoded by significantly more genes than the other capsule groups (S1 Fig). Whereas the median Group I and ABC-dependent systems had between 19 and 16 genes, the synthase-dependent HAS (hyaluronic acid) capsule was encoded in three genes and the Syn_CPS3 in four (S4 Table). These differences may be affected by the abovementioned inaccuracies in capsule loci delimitation and by the definition of the models. Nevertheless, our results show that some groups of capsules have loci of almost invariable sizes (all Group IV capsules), whereas others showed very significant variation in the number of components (especially Group I and ABC, see lower slopes in S1 Fig). These results give statistical support to the idea that the number of capsule components differs markedly between groups.

We then searched to test if genome size was correlated with the number of genes encoding a capsule system. Genomes encoding capsule systems were generally larger than those lacking them (Wilcoxon rank sum test, P < 0.001), but the number of genes in the capsule loci showed no correlation with genome size when controlled for phylogenetic dependence (S4 Table). This suggests that constraints on genome size have no significant effect on the complexity (number of genes) of each capsule system.

### Frequent and non-random co-occurrence of capsule systems

**Fig 3:**
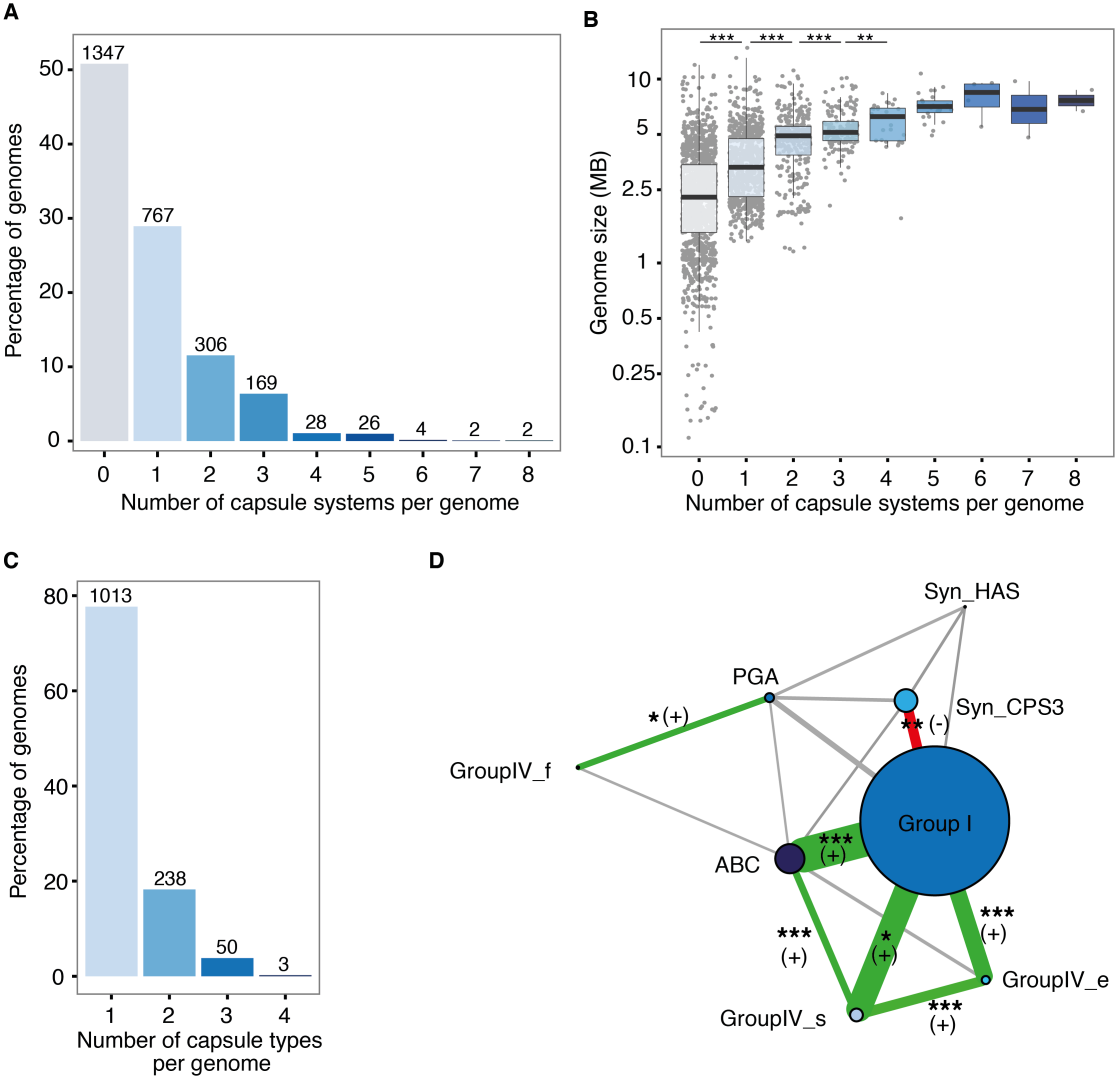
Co-occurrence of capsule systems in genomes. **A**. Percentage of genomes with none, one or more capsular systems, irrespective of capsule groups. The number of genomes is indicated on top of the bars. **B**. Box-plots of the distribution of genome size in respect to the number of capsule systems identified in the genome. Y-axis is in log scale. Each point reflects one individual genome. The boxes span from the first to third quartile and the central line indicates the median. The length of the vertical line extends from the first and third quartile to the lowest and highest data points that are no more than 1.5 times away the interquartile range. Genome size was calculated as the sum of all chromosome and plasmid sizes, and was log_10_-transformed prior to statistical analysis. Asterisks indicate significant differences in median genome size across groups, Tukey *post hoc* test. *** *P* < 0.0001, ** *P* < 0.01. **C**. Histogram of the number of capsule groups per genome (among genomes encoding at least one capsule). The number of genomes is indicated on top of the bars. **D**. Network of co-occurrences of capsule groups. The size of the nodes is proportional to the number of genomes encoding the capsule group. The width of the links is proportional to the total number of co-occurrences. Red (-) and green (+) edges indicate significant negative and positive associations, respectively. We indicate three types of significant results. Main results are for the test of significant dependent evolution between capsule groups. These pass two tests: a test of independence on a contingency table (χ^2^) and a test of phylogenetic dependence accounting for phylogenetic uncertainty (see Methods). *** *P* < 0.0001, ** *P* < 0.01, * *P* < 0.05.

We found that almost half of the genomes encoding capsules have more than one system (40%, Fig 3A). Strikingly, two environmental cyanobacteria encoded up to eight capsules, and 23 other species encoded between five and seven systems (S5 Table for details). Among these 25 species, all with large genomes (>4.5 Mb), we identified very few human-associated bacteria: a commensal Bacteroidetes, and some opportunistic pathogens of the *Burkholderia cepacia* complex. Instead, most of the 25 genomes were from mutualistic or environmental bacteria, including several α- and β-Proteobacterial rhizobia. The size of the genome was correlated with the number of capsules it encodes (Spearman’s rho = 0.16, *P* < 0.0001 after phylogenetic correction) (Fig 3B), and with the sum of all capsule components (Fig 3B, and S4 Table for phylogenetic corrections). Hence, while the number of genes in a capsule system is not associated with genome size, larger genomes tend to encode more capsule systems, and thus have more capsule-associated genes.

Nearly half of the genomes with multiple capsule systems encode several occurrences of the same capsule group (246 out of 537). We analyzed their sequence similarity to test if they could have arisen by recent large segmental duplications. The systems were typically very divergent: 97% of the intra-genomic comparisons showed less than 80% sequence similarity at the homologous proteins used to identify the group (see Methods). Systems of the same group were not found in tandem, as expected if they had resulted from recent duplications [41] and only eight (out of 1004) pairs of consecutive systems were less than 10 kb apart (S2 Fig). Furthermore, some genomes encoded two (238), three (50), and up to four (in *E*. *coli* strain REL606) different capsule groups (Fig 3C). Hence, multiple capsule systems do not seem to originate from recent segmental duplications.

Remarkably, more than half of the genomes encoding an ABC-dependent capsule also encode a Group I capsule (S6 Table), and all genomes coding for Group IV_s and Group IV_e capsules also code for at least one other capsule group. A non-random assortment of capsule groups would suggest epistatic interactions between capsules. To test this possibility, we analyzed the co-occurrence of capsule groups in the light of the underlying phylogenies (see Materials and methods). We used Pagel’s method [42, 43] to fit models of dependent evolution between capsule groups and compared them with models assuming independent evolution (see Methods). We observed significant co-occurrence of Group I capsules and most of the other capsule groups (Fig 3D and S7 Table), including ABC-dependent capsules and Group IV_s. We also observed frequent co-occurrence between PGA and Group IV_f capsules (Fig 3D and S7 Table). In contrast, several groups of capsules showed unexpectedly low co-occurrence patterns suggesting the existence of negative epistatic interactions. For example, we only identified two co-occurrences of Group I and Syn_HAS.

### Capsule co-occurrence within the Enterobacteria

**Fig 4:**
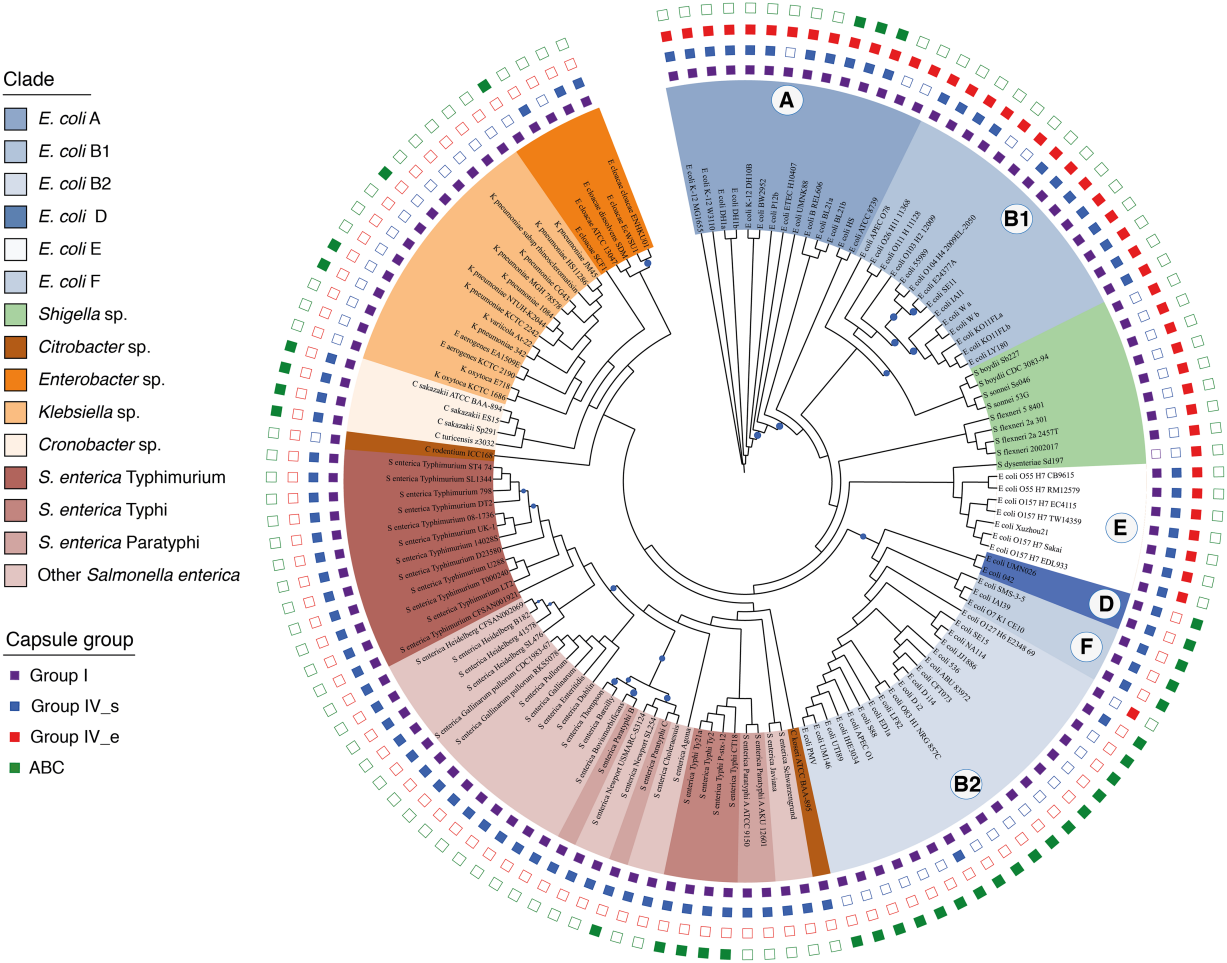
Cladogram of selected enterobacterial species and the capsule groups detected in their genomes. The tree was built using the protein sequences of the 759 families of the core genome of Enterobacteria. Squares on the outer part of the tree indicate the presence (full) or absence (empty) of different capsule systems in the corresponding genomes. Background colour indicates the phylogenetic group of each genome. The size of the circles along the branches are proportional to bootstrap values ranging between 20 and 99. Absence of circles indicates a bootstrap of 100%.

The family of Enterobacteria showed the most frequent co-occurrence of capsules from different groups and subgroups (Fig 4, see S8 Table for the complete list of genomes). Since it also includes several of the model organisms used to study the capsule—*E*. *coli*, *S*. *enterica*, *K*. *pneumoniae*–we analyzed these genomes more in detail. We detected seven out of the 24 different combinatorial possibilities offered by the four different capsule groups identified in the clade. In the line of the results mentioned in the previous paragraph, we observed a clear pattern of correlation between Group IV_s and Group I capsules in enterobacterial genomes (Fig 3).

We observed that closely related genomes often encode different capsule systems. For instance, within the phylogenetic group B1 of *E*. *coli*, the two enteroaggregative pathotypes (*E*. *coli* 55989 and *E*. *coli* O104) encode the same capsule groups, which differ from all the others of the same phylogroup. Similarly, the two only commensal strains (ED1a and SE15) of the phylogroup B2 share the same capsular combination, which is different from all other B2 genotypes (S8 Table). Finally, *E*. *coli* from phylogroup A, comprising a majority of commensal bacteria, often have at least three different capsule groups, which is significantly more than other clades including many pathogens, such as *Shigella sp*. and *E*. *coli* B2 (two capsule systems per genome, on average). These results revealed an association between capsule groups and bacteria-host interactions. To conclude, the rapid genetic turnover of capsule systems within closely-related genomes [44] suggests that they can rapidly change to face environmental or lifestyle changes.

### Capsules are rare in obligatory and frequent in facultative pathogens

**Fig 5:**
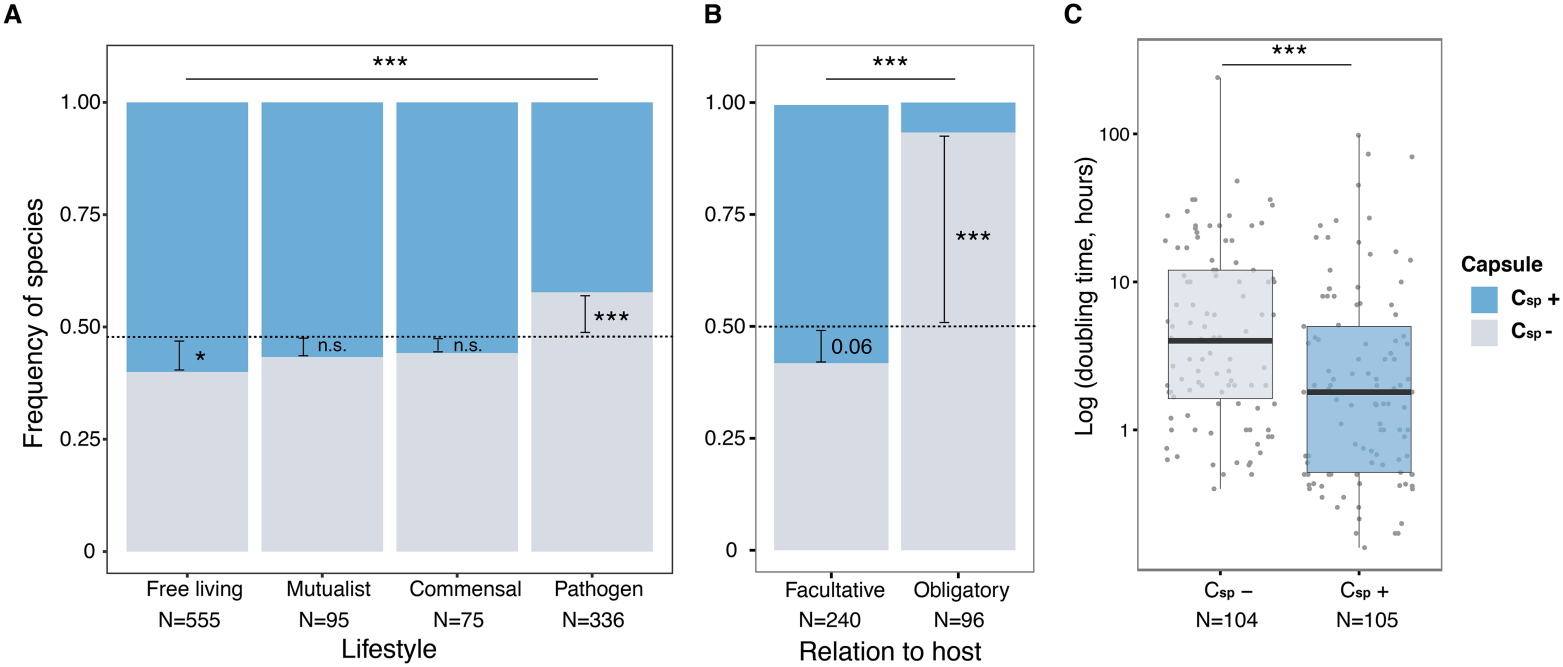
Association between the presence of capsules and lifestyle traits. Frequency of bacterial species with and without detectable capsule system in function of their lifestyle (**A**) and level of host-association (**B**). Two different statistical assays were performed. Stars inside bars represent the result of two-tailed binomial tests to measure the difference between the observed over the expected events, indicated by the dashed line corresponding to the database bias. The *P*-values shown on top of the bars represent the result of the test of dependence between the two traits corrected by the phylogeny as calculated by the *fitPagel* function included in the phytools package for R. **C**. Minimum doubling time across C_sp_+ and C_sp_- bacteria. Statistics correspond to the test of a significant difference between the two groups (Wilcoxon rank sum test). ** *P* < 0.01 *** *P* < 0.001. It was controlled for phylogeny using the *compar*.*gee* function. The controls for phylogeny were done (in panels A, B, and C) using 100 trees obtained by bootstrap experiments to account for uncertainty in the phylogenetic reconstruction. The distributions of the corresponding 100 *P*-values are provided in S5 Fig. (see Methods).

The observation that multiple capsules are more frequently observed in commensals, mutualists or environmental bacteria seems at odds with the hypothesis of a tight association between capsules and pathogenesis. We classified bacterial species according to the degree of host-association they commonly exhibit (S1 Dataset, see Methods for criteria and [45, 46]) and found that the probability of encoding a capsule depends on the lifestyle of the bacteria (Fig 5A), even when accounting for genome size (S9 Table). We then first tested whether free living species were more likely to code capsules than pathogens. We found that, indeed, capsules were slightly rarer in pathogenic species as opposed to free living species (Fig 5A and S4 Fig). The lower frequency of capsules in pathogens remains qualitatively similar when commensals and mutualists (or both) are grouped together with free living species. Additionally, we observed no difference in genome size between pathogenic bacteria encoding a capsule system and the others, suggesting that the association between the presence of capsule and pathogenesis is independent of genome size.

Many of the pathogenic bacteria in our dataset are facultative or opportunistic. These bacteria typically have environmental reservoirs and larger genomes than obligatory symbionts (pathogens or mutualists) [47, 48]. We observed that many facultative pathogens encode capsules, in contrast to most obligate pathogens, independently of the differences in genome size between the groups (Fig 5B and S1 Dataset). The difference between obligatory and facultative pathogens remained statistically significant when controlling for phylogenetic structure (see Methods, Fig 5 and S5 Fig). Whereas very few obligate pathogens encoded a capsule, amongst which *Shigella flexnerii* and *Mycoplasma mycoides*, a small majority of the facultative pathogens encoded a capsule (Fig 5B). This result does not change qualitatively when only human pathogens are taken into account.

Facultative pathogens tend to start infections only at high infectious dose (ID_50_), to be motile, and to grow fast under optimal growth conditions [49]. These characteristics also tend to be associated with a lack of ability to kill professional phagocytes of the immune system or to survive in the intracellular milieu of these cells [49]. Since capsules may provide some resistance to phagocytosis, we enquired on the possible association between the capsule, minimum doubling time, and ID_50_ (measured in humans as available for only 39 species, [49]). We observed that bacterial species that encode a capsule system (C_sp_+), show significantly lower minimum doubling times (Fig 5C and S4 Fig), higher ID_50_, are more likely to be motile, and are less likely to be able survive phagocytosis than those that do not encode a capsule (C_sp_-) (Fig 5, S3 and S4 Figs). Whereas the first association was significant even when controlling for genome size and pathogenicity (S9 Table), and phylogenetic dependence (S5 Fig), the two latter associations were not statistically significant due to lack of statistical power (there is little data available for these traits). Overall, our results indicate that capsules are more readily associated with facultative pathogens with high infection doses and short minimal generation times.

### C_sp_+ bacteria are over-represented across different environments

**Fig 6:**
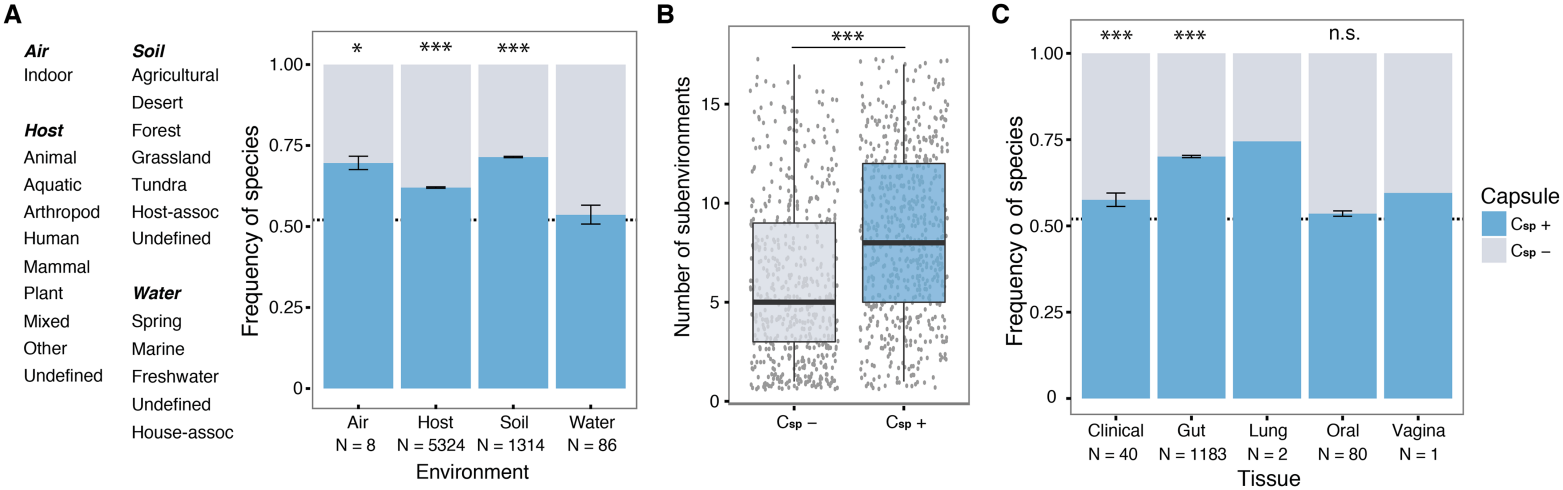
Distribution of C_sp_+ in the environment. **A**. Detail of the different subenvironments analyzed. Frequency of species with and without capsule systems per environmental category (averaged across metagenomes) depends on the environment (χ^2^ = 18.5, *df* = 3, *P* = 0.0004). The dashed line indicates the frequency of C_sp_+ in the genome database. * *P* < 0.05, *** *P* < 0.001 for significant difference from the expectation of 0.52, Wilcoxon sing rank test, with Benjamini & Hochberg *post hoc* correction. Error bars indicate standard error. **B**. Distribution of the number of different environmental subclasses where C_sp_+ and C_sp_- were found. *** *P* < 0.001 Wilcoxon test. **C**. Frequency of C_sp_+ and C_sp_- depends on the body location (χ^2^ = 16, *df* = 4, *P* = 0.003). C_sp_+ are significantly overrepresented in clinical samples and in the gut. Statistics could not be performed for the lung and vagina as only two and one metagenomes were available, respectively. Statistics as for part A.

We analyzed microbiome data to confirm that capsule systems are frequent in environmental bacteria and in facultative pathogens (that often have environmental reservoirs). Unfortunately, loci encoding capsule systems are too long and complex to be identifiable in the sequences of metagenomes. To circumvent this difficulty, we identified the presence of the species for which we had at least one complete genome in a large number of publicly available metagenomics datasets (16S rRNA). We used this information to quantify the abundance of each species and, using the species' complete genomes as a proxy, to predict the presence of capsules in these environments. Specifically, we searched for the presence of C_sp_+ in 16S datasets from four classes and numerous sub-classes of environments (Fig 6A). This allowed both the qualitative and quantitative identification of bacterial species in 6700 environmental 16S datasets (S8 Table, see Methods). We computed the abundance of C_sp_+ relative to C_sp_- species in the 16S datasets in qualitative (number of species) and quantitative (number of 16S sequences) ways (see Methods). The percentage of C_sp_+ was similar in the 16S (53% out of 1197 bacterial species) and in the genome (52%) datasets. C_sp_+ were more frequently present and quantitatively more abundant than C_sp_- in all four classes of environments, even if this trend was not always significant (Fig 6A and S6A Fig).

Capsules allow Prokaryotes to withstand a series of stresses, from environmental disruptions to protozoa grazing, and are expected to be associated with broader environmental ranges. Indeed, C_sp_+ species were present in significantly more environmental subclasses than C_sp_- (Fig 6B). Importantly, the number of environmental subclasses for a given species increased with its average number of capsules per genome (Partial Spearman test, *P* < 0.001, after correction for genome size). These results show that bacteria encoding capsule systems are able to colonize a larger variety of environments.

### C_sp_+ in the human microbiome

The vast majority of previous studies focused on capsules of bacterial pathogens. To disentangle the relation between capsule and pathogenesis, we analyzed the presence of C_sp_+ species in human-associated datasets. We first checked that we were able to identify well-known pathogens in the host-associated environments. Indeed, we detected pathogens with Group I capsules, such as *K*. *pneumoniae* and *S*. *pneumoniae*, as well as pathogens with ABC capsule systems, namely *Neisseria meningitidis*, in samples of the human microbiome, and sometimes also in other environments (S10 Table). The total abundance of species encoding capsules within the human host varied between body locations (Fig 6C), and was higher overall than within the complete genome database (57%, binomial test, *P* = 0.005). C_sp_+ species were more abundant than C_sp_- in all locations, and especially in the gut microbiota, which encompasses the largest fraction of bacteria in the human body. Likewise, clinical samples over-represented C_sp_+ species. Interestingly, we observed that the relative abundance of C_sp_+ and C_sp_- was strongly dependent on the human body sites (ANCOVA, P < 0.001, S6B Fig).

Taken together, our results show that even if capsules are relatively rare among obligatory pathogens, they are very frequent in human microbiota where they are frequently associated with clinical conditions.

## Discussion

### Unraveling the repertoire of capsules of Prokaryotes

Capsules play important roles in bacterial virulence, but their study has been hampered by the lack of computational tools to identify them in genomes. Our tool, CapsuleFinder, identifies the eight major groups and subgroups of capsule systems in bacterial and archaeal genomes and is thus complementary to software designed to analyze very specific capsule systems, *e*.*g*., the recently released Blast-based tool to identify capsular serotypes in *Klebsiella spp*. (Kaptive, [50]). The models in CapsuleFinder can be modified to either increase specificity (obtain systems closer to the experimental models) or sensitivity (to detect more distantly related systems). This can be done by changing the number, type and genetic organization of the components that are required to identify a system. Users can also add novel models and protein profiles to improve the tool, *e*.*g*., to account for novel experimental data. If enough experimental serotype data is available for a given species, then the models can be specified in order to infer a putative serotype for the strains.

The construction of our models was based on previous experimental studies restricted to a relatively small number of model organisms from Proteobacteria and Firmicutes. Capsules, like many extracellular structures [51], are subject to rapid evolution and reorganization via recombination, complicating their detect from a small number of taxonomically restricted reference systems. In spite of this, we were able to identify them in many phyla of Prokaryotes—even in Archaea—with few putative false positives. Hence, we expect to have identified the majority of capsules of known groups in the complete genome database. The entire collection of capsule systems can be consulted in our database (http://macsydb.web.pasteur.fr/capsuledb/_design/capsuledb/index.html and S1 Dataset). Further, the identification of capsule systems by CapsuleFinder opens the way for their comparative analysis, including the study of how horizontal transfer leads to serotype switching across bacteria [52, 53].

### The abundance and diversity of capsule systems

Our analysis showed that a majority of Prokaryotes encodes at least one capsule system (Fig 2). Group I, PGA and Syn_CPS3 are the most widespread across the Bacteria whereas other groups were restricted to a few taxa, namely Group IV and Syn_HAS. Importantly, we found capsule systems in all phyla for which more than ten genomes were available. Future work will be necessary to assess if poorly sampled phyla—Chrysiogenetes, Deferribacteres, and Elusimicrobia—are effectively devoid of known capsule groups or if they encode novel groups of capsules. It will also be interesting to analyse capsule prevalence in newly discovered uncultivable phyla characterized by single-cell genomics since they may reveal novel capsule groups (or variants of existing ones) [37, 54]. Given our results in the phyla with higher representation in the database, capsules might occur across all prokaryote phyla.

Capsule-like structures have been described in Archaea [38–40], where a previous bioinformatic study revealed the presence of proteins similar to those involved in the synthesis of the PGA capsule in one species [29]. We identified PGA capsule systems and also Syn_CPS3 systems in many genomes of Archaea. These two groups of systems have few components and we couldn't find data suggesting that they allow extensive serotypic variation. However, the lack of more complex capsule groups, should be subject to caution owing to the lack of experimental data. Furthermore, our tools to identify capsules were based on bacterial systems. Alternatively, the peculiarities of the cellular envelope of Archaea may explain the absence of certain capsule groups in the phyla. Most Archaea have a S-layer composed of glycans that might affect secretion or cell surface association of certain capsules.

### Capsule multiplicity

Our method may underestimate the number of capsule systems of the same group co-occurring in a genome owing to strict localization rules in our models to avoid false positives. For example, not all Group I *Bacteroides thetaiotaomicron* were detected because some operons lacked the minimum mandatory genes required to identify the gene cluster as a capsule system (S3 Table). This suggests that some structural elements involved in capsule secretion might be shared between different systems. Yet, to date, the existence of genomes encoding multiple capsules of the same group had previously been documented in only a few species, namely *Bacteroides spp* [55, 56]. In *B*. *fragilis*, a key commensal of the gut microbiome, there are several Group I capsule systems, some of which are implicated in the formation of intra-abdominal abscesses [57]. This species encodes a DNA inversion mechanism that combinatorically switches the expression of the different systems [58], producing extremely diverse capsule structures that are thought to increase bacterial fitness in the intestinal milieu by virtue of their immunomodulatory properties [59]. In this case, capsule variation seems to evolve as a response to the rapid change of the human immune system [58].

Bacteria may also encode multiple capsules from different groups, as described for the PGA and Syn_HAS capsules encoded in different plasmids of *Bacillus cereus* biovar *anthracis*[60]. Co-expression of different capsule groups is thus possible, implicating that capsules will physically interact in the cell envelope. Our data suggests that capsule combinations can be even more complex, since this same strain encodes a Group I capsule in the chromosome, and some enterobacteria encode up to four different groups of capsules.

The non-random patterns of co-occurrence of different capsule groups observed in this study suggest that capsule repertoires are affected by epistatic interactions (Fig 3D). The nature of these interactions depends on whether the different capsules are expressed at the same time, thereby producing combinatorial diversity, or at different moments, *e*.*g*., in response to different environmental cues. Positive epistasis may result from the synergistic combination of the properties of the different capsules, *e*.*g*., different capsules may provide a broader range of environmental protections and capsule switching (or variation in the proportions of each capsule group) may facilitate escaping grazing protozoa, professional phagocytes of the immune system, or bacteriophages. Negative epistasis associated with co-expressed capsules may result from problems in accommodating different capsule structures in the cell envelope. Negative epistasis between capsules that are not co-expressed could be caused indirectly by the effects of the genetic background, *e*.*g*., because some groups of capsules are more compatible with certain membrane structures (pili, flagella, secretion systems) than others.

The mechanisms leading to the acquisition of multiple capsules will have to be studied in detail in the future, but our results already provide some clues. We observed that many genomes encode capsules of different groups, that capsules of the same group are very divergent in sequence and are encoded in distant regions in the genome (or in different replicons). This suggests that capsules were independently acquired by multiple events of horizontal gene transfer. This fits the abundant literature showing that capsules vary rapidly within species by recombination and horizontal transfer [61–63]. It also explains why most capsule systems are encoded in a single locus, since this facilitates transfer [64]. Finally, the outcome of capsule transfer is likely to depend on the environmental challenges faced by the bacteria and will be affected by the abovementioned epistatic interactions.

### The capsule increases environmental breadth

A substantial part of the previous literature on capsule systems has focused on bacterial pathogens and on the role of capsules as virulence factors. For instance, it has been shown that acquisition of certain capsule types by horizontal gene transfer in *Neisseria meningitidis* allowed the bacteria to increase in pathogenicity and going from non-pathogenic carriage to infectious state [52, 53]. It was thus surprising that non-pathogens are more likely to encode capsules, and that, among pathogens, the ones establishing obligatory antagonistic interactions with their hosts typically lacked a capsule.

The abundance of capsules across most phyla and environmental classes, and their rarity among obligatory pathogens, suggest they play important roles beyond pathogenesis. Indeed, the capsule also constitutes an advantage for commensal bacteria of the gut. To colonize the gut, the bacteria have to first withstand the harsh conditions of the stomach and then grow and multiply in the duodenum and colon, in the presence of bile salts. In *Bifidobacterium longum*, capsule expression would enhance survival in the stomach and allow growth under high concentrations of detergent-like bile salts in the duodenum [65]. Similarly, a study performed in yeast has shown that although capsules from environmental and pathogenic strains display similar composition and features, they fulfil different roles [66].

Capsules are an example of the ability of bacteria to evolve structures serving multiple purposes in different environments. Like other virulence factors, such as some iron capture proteins, while evolving as an adaptation to an environment they also confer an advantage during pathogenesis (exaption), either during colonization or transmission across hosts [47, 67].

Our data also shows that the presence of capsule systems, and especially multiple systems, is associated with broader environmental ranges. The ability to express different capsules, or combinations of them, can result in heterogeneity in the surface charge of bacterial cells which can in term influence important phenotypes such as cellular adhesion to tissues or surfaces, susceptibility to certain cationic peptides, etc. In the aforementioned *B*. *cereus* strain, the co-expression of the two capsules did not increase virulence in two different animal models, but rather favoured bacterial colonization and dissemination [60]. Similarly, previous studies in soil-borne nitrogen-fixing bacteria indicated that bacterial exopolysaccharides and lipopolysaccharides that can be similar to capsules are involved in species-specific interactions between the bacteria and the host [68]. This is consistent with our observation that capsule multiplicity increases environmental breadth, and suggests that it may also increase host range.

Taken together, our study revealed an unsuspected prevalence of capsules in Prokaryotes, especially in environmental bacteria and facultative pathogens. Our results are in line with the multitude of roles proposed for capsules and are not consistent with the idea that capsules evolved to facilitate pathogenesis. Instead, they highlight that capsules might have an important role in facilitating bacterial adaptation to novel or changing environments. Interestingly, we found many capsule systems in soil bacteria, from which probably originated capsulated opportunistic multi-resistant bacteria such as *Klebsiella pneumoniae*, *Enterococcus faecium*, and *Acinetobacter baumanii* [69–72]. Capsules may have thus evolved primarily as an adaptation to a range of different environments, and this facilitated subsequent ecological transitions towards host colonization and pathogenesis.

## Materials and methods

### Data

#### Genomes

We analyzed 2786 chromosomes and 2087 plasmids of 2484 bacterial and 159 archaeal fully sequenced genomes from NCBI RefSeq (ftp://ftp.ncbi.nih.gov/genomes/, downloaded in November 2013). This accounts for 1440 different species of which 140 are Archaea. The details are listed in S1 Dataset.

#### Metagenome samples

We downloaded 6743 metagenome samples (16S rRNA assembled reads) and associated metadata from MG-RAST (http://metagenomics.anl.gov/, last accessed on March, 2016). The identification of capsules was performed at the genome level whereas metagenome and life-style analyses were performed at the species level. Analysis at the species level required a classification of species into those encoding capsules (C_sp_^+^) and those lacking them (C_sp_^-^). In the vast majority of cases, the different strains of a species had the same group of capsules. When they didn't, we used the following procedure. If the species had between 2 and 4 genomes, we excluded the species if some genomes encoded capsules whereas other lacked them. If the species had more than 4 genomes, we accounted for the frequency of the rare variant. When at least 80% of the species concurred (in presence or absence of the capsule) they were classed according to the predominant trait. Otherwise, we excluded the species. This led to the exclusion of a very small number of species (25 species, less than 2% of the total).

#### Pathogenesis and host-association

Classification of bacteria in terms of pathogenicity is difficult because the ecology of most species is not well known and some bacteria have lifestyles between commensalism and pathogenicity. There is a very large literature on human pathogens that makes the distinction between commensals/free living and pathogens relatively simple, once one decides that the existence of a strain producing frequent infections in humans is enough to class the species as a pathogen. Whenever possible, the information related to pathogenesis was retrieved from the Bergey's Manual of Systematic Bacteriology [73] to maximise the homogeneity of the criteria. When needed, we used primary literature (references in S1 Dataset). We did not class some species because information was lacking or was ambiguous. When the classification was based on little information we added a comment in the table with appropriate references. Bacteria were classed as **facultative** or **opportunistic** *pathogens* when there were several reports showing their role in host infection, but they are also commonly able to proliferate as commensals or as free-living bacteria. Most nosocomial pathogens fall in this category. Bacterial species were classed as **obligate pathogens** when they are known to require infection to proliferate significantly in the host. This is the case of many pathogens that are intra-cellular, have small genome size, or are uncultivable in synthetic medium, *e*.*g*., many *Chlamydia*, *Mycoplasma*, and *Rickettsia*. Bacteria were classified as **free-living** when no host has been described and when there were no—or very few—reports of infections caused by the strains of the species (mere presence in clinical isolates was not enough to define it as a pathogen). This class includes many soil- or water-associated bacteria, as well as nearly all extremophiles. Bacteria were classed as **commensal** when they were not classed as pathogens, and were described as typically host-associated but lacking clear evidence of establishing mutualistic relationships. Many bacteria from the healthy human microbiome bacteria fall in this category. Bacterial species were classed as **facultative mutualists** when there is evidence of mutual beneficial association with the host. This includes rhizobial bacteria, for instance. Finally, bacteria were classed as **obligatory mutualists** when there is evidence of mutual beneficial association with the host and their growth strictly depends on the host. This is the case of many endosymbiotic bacteria, like *Buchnera*. The goal of this classification was to identify pathogens. Hence, a species with many commensal strains and some pathogenic ones, was classed as a facultative pathogen.

We also described the host-association. While host-association with poorly studied Eukaryotes may be hard to assess, the main interest of this work was on human pathogens that are usually well described. The class "human" includes bacteria frequently found in humans (and eventually also in other Eukaryotes). The class "other mammals" includes bacteria growing in association with mammals but not usually found in humans. The class "other animals" corresponds to bacteria that grow on animals, but not usually found in mammals. The class "plants" corresponds to bacteria usually associated with plants, like rhizobia or phytopathogens. The class "other" corresponds to bacteria associated with Eukaryotes (typically with protozoa, like amoeba). This classification is hierarchical (humans > other mammals > other animals > plants > other), *i*.*e*., a bacteria present in hosts of all these groups is classed in the top group (humans).

The details of the classification are listed in S1 Dataset. The histograms with the distribution of the different classes are in S7 Fig. From this table we used two sets of categories. The "Pathogen" category includes the facultative, opportunistic pathogens and the "No Pathogen" includes the free-living but not the mutualists. In terms of relation with the host, we used only pathogens, and compared facultative and opportunistic pathogens with obligatory pathogens.

### Models for extracellular capsules

We built a model for each group of capsule with the information we could obtain from the literature. We specified models with mandatory (biologically essential components for a putative functional system, a majority of which, if not all, are required to identify and classify the systems), accessory (non-essential components used to improve the annotation of the system), and forbidden components (*e*.*g*., those found in other capsule groups and not in the focal one, thus helpful to discriminate between the capsule groups, see below the example of Group IV capsules). Of note, due to the low conservation of some mandatory elements, for example Wzy polymerases, in some instances a system could be validated even if a certain number of mandatory components were not detected. This is controlled by the option *min_mandatory_genes_required*. The parameters used for the minimum quorum of mandatory genes were set based on the analysis of experimental systems and on our previous experiences with the development of similar models for protein secretion systems and CRISPR-Cas systems [33, 36]. While these systems are very different, they have in common that certain components that are thought to be biologically necessary may not be identifiable by sequence analysis either because they evolve too fast, or because they can be replaced by analogues lacking sequence homology.

Additionally, we specified that components should be encoded in a single locus (defined as a series of genes respecting a maximal pre-specified distance between consecutive elements). When the available experimental data suggested that it was relevant to allow components to be encoded elsewhere in the genome, we defined them as *loners* in the models. Models were written in plain text, using a specific XML grammar, and can be modified by the user (see http://macsyfinder.readthedocs.io/en/latest/ for details). For simplicity, we named the components after the protein names in the species that served as a biological model for each group of capsule. The names of the homologs to these proteins in other species with experimentally validated systems are listed in S1 Table. Polymer-specific enzymes were regarded as accessory in the models because they can be homologous to enzymes of other cellular processes [18].

#### Group I or Wzx/Wzy-dependent

Group I capsules rely on the action of the Wzy polymerase and the Wzx flippase [18, 20]. Because Group I capsules have different components in monoderms and in diderms, we built two distinct models (Fig 1A and S1 Table). The model for diderms was based on the biological model of *E*. *coli* K30 [74] and requires three proteins (Wza, Wzb, and Wzc). The model for monoderms was based on the common elements of several *S*. *pneumoniae* serotypes (9, 12, 14 & 15) [11] and requires at least four other proteins (Wzd, Wze, Wzg, and Wzh). Because Wzx and Wzy are often poorly annotated and poorly conserved within and across species, we accepted systems that lacked one of the five biologically mandatory components when a minimum of 6 proteins (including polymer-specific enzymes) were present. In spite of the existence of a profile for Wzy in PFAM 28.0, we built a new one based on experimentally validated proteins because our preliminary analysis showed that the PFAM profile missed several experimentally validated systems (S1 Table). The results reported throughout the text take into account the capsules of this group in both monoderms and diderms. The division of monoderms and diderms was made exclusively at the level of the identification of the system to increase the accuracy of the method.

#### ABC-dependent (groups II and III)

These capsules are synthesized by an ATP-binding cassette (ABC) transporter of type 2, composed of two proteins, a nucleotide-binding protein, KpsM, and a transmembrane protein, KpsT [20] (Fig 1B and S1 Table). We built one single model for group II and III capsules (Fig 1B), because their key components are the same, their genetic organization seems very similar, and experiments have shown that the major differences between the groups are at the level of gene regulation [20]. The model for these groups was based on the system of *E*. *coli* 536 [75]. Diderms require two more proteins than monoderms for capsule secretion: KpsD (homologous to Wza of Group I), and KpsE (an adaptor protein). Two other proteins are needed for capsule export in *E*. *coli* 536—KpsC and KpsS [20]—but their precise function is unknown and they are dispensable in other well-studied ABC-dependent capsules like those of *Actinobacillus pleuropneumoniae* [76] (Fig 1B). We therefore decided to include KpsM, KpsT, KpsD and KpsE as mandatory and KpsC and KpsS as accessory proteins (Fig 1B).

#### Group IV (subgroups e, f and s)

This heterogeneous group of capsules, also named O-antigen capsules, depends on the Wzy polymerase. Each subgroup differs in all other components and their genetic organization (Fig 1E). Thus, we defined one model for each subgroup (Fig 1E). To avoid false positives and better discriminate between Group IV and Group I capsular groups, we defined Wzx flippase as a forbidden component. Additionally, ABC transporters, exclusive ABC-dependent capsules were also included as forbidden elements. The *E*. *coli* E2348/69 0127:H6 Group IV_e capsule cluster is made of a seven-gene operon with four outer-membrane lipoproteins with β-barrel domains (YmcA-D) of unknown function and the secretory components homologous to Wza, Wzb and Wzc of Group I capsules. All these components are required for the production of the capsule [27]. The *Francisella tularensis Schu S4* Group IV_f gene cluster is composed of a glycosyltransferase (family 8), a Wzy-like polymerase and a third gene of unknown function but described as essential [77]. We did not include *dnaJ* and *hemH* genes in our model because they are encoded elsewhere in the genome and affect capsule production by an unknown mechanism [78] most likely due to indirect epistatic interactions rather than direct implication in capsular biosynthesis. The *S*. *enterica* serovar Typhimurium LT2 Group IV_s system has ten genes, of which three were shown to be essential and are thus mandatory in our model [26]. To minimize false positives, our model also required the presence of at least two of the non-essential genes (accessory components, Fig 1E).

#### Synthase-dependent subgroups (Syn_CPS3, Syn_HAS)

Syn_CPS3 and Syn_HAS synthase-dependent capsules rely on the activity of CpsS and HasA respectively, which are the processive glycosyltransferases that polymerize and secrete the capsule (Fig 1C). The model for the *Syn_CPS3* subgroup was based on the 5-gene CPS3 operon of *Streptococcus pneumoniae* serotype 3 [79], and the model for the Syn_HAS subtype on the 3-gene hyaluronic acid operon of *Streptococcus pyogenes* [28, 80]. Aside from the processive glucosyltransferase, our models require the presence of other sugar-modifying enzymes commonly associated to subgroups Syn_CPS3 (three components) and Syn_HAS (two components). These polymer-specific enzymes are required in the model and exchangeable across capsule subgroups (Fig 1C). To minimize false positives, we defined that a putatively functional system required the presence of a processive glycosyl transferase of the subgroup, and a minimum of two other components. Some mandatory components of Group I and ABC- capsules were included as forbidden in the model, to improve discrimination from these two other capsule groups.

#### Proteic capsule (poly-γ-d-glutamate, PGA)

The model for the PGA group was based on the PGA synthesis operon of *Bacillus anthracis* [29] composed of five genes (CapABCDE). The cloning of *capABC* alone into *E*. *coli* resulted in the production of PGA [81]. Other studies mention that PGA production can occur even in the absence of CapA [82]. In fact, CapA, in conjunction with CapE, seem to fulfill a regulatory role and is not essential in some bacteria [83]. We therefore defined CapB and CapC as mandatory and CapA and CapE as accessory. CapD was allowed to be encoded apart from the other components (it is thus a *loner* in our model, Fig 1E). We defined PgsS, a PGA-digestive enzyme, as forbidden, because this enzyme releases the polymer to the environment and therefore cannot be associated with a capsule system (Fig 1D).

#### Limitations of the models

The definition of tools to identify capsules is complicated by the small number of experimentally studied capsule systems (and their concentration in a small number of phyla). For example, we could not find any reports of ABC capsules in monoderms [18], and a search for the numerous ABC transporters in *Firmicutes* showed that none branched with the known ABC components involved in capsule secretion. We therefore preferred to remain cautious and restricted our analysis of ABC-dependent capsules to diderms. We were unable to build a model for the *tts* synthase-dependent capsule described only in *S*. *pneumoniae* serotype 37 [84], because we could not find the 5-aminoacid long degenerate motif that specifically discriminates the *tts* processive glycosyltransferase from other non-processive glycosyltransferases. The discrimination between capsule systems and other systems dedicated to the synthesis of EPS is simpler when the capsule is encoded in one single locus alongside other essential components of capsule synthesis and in most cases this restriction was integrated in our models (see Materials and methods). This is by very far the most common genetic organization described in the literature, but exceptions have also been reported (*e*.*g*., in *Porphyromonas gingivalis* [85]). Finally, diderms require at least one more protein than monoderms to enable translocation through the outer membrane. We therefore built independent models for diderms and monoderms.

### Identification of capsule systems

We used MacSyFinder to search for capsule systems [33]. This program takes as input a proteome, a set of hidden Markov models (HMM) protein profiles (one for each component of the system, see below), and models describing the number of components and their genetic organization (Fig 1). MacSyFinder identifies the individual components of each capsule system using hmmsearch from the HMMER package v3.1b2 [86]. A component was retained for further analysis when its alignment covered more than 50% of the length of the profile and obtained an e-value smaller than 0.001.

### Definition of HMM protein profiles

We used 58 different HMM protein profiles in our searches (S1 Table), 31 retrieved from the PFAM 28.0 database (http://pfam.xfam.org, [87], last accessed November 2015) and 27 built in this study. Each protein profile was constructed as follows (except when explicitly stated otherwise). We started from a well-described and experimentally-validated component of a system and used BLASTP v 2.2.28 [88] (default settings, -v 4000, e-value < 10^−4^) to search for homologs among complete genomes. To reduce the redundancy of the dataset (*i*.*e*., to remove very closely related proteins), we performed an all-against-all BLASTP v 2.2.28 analysis and clustered the proteins with at least 80% sequence similarity using SiLiX v1.2.9 (http://lbbe.univ-lyon1.fr/SiLiX, default settings) [89]. We selected the longest sequence from each family as a representative. The set of representative sequences was then used to produce a multiple alignment with MAFFT v7.215 using the L-INS-i option and 1000 cycles of iterative refinement [90]. The alignment was manually trimmed to remove poorly aligned regions at the extremities, using SEAVIEW [91]. The HMM profile was then built from the trimmed alignment using hmmbuild (defaults parameters) from the HMMER package v3.1b2 [86].

### Model validation

We validated the method to identify capsule systems using two published lists of capsulated bacterial pathogens [19, 35]. Since these lists were very short, and not necessarily meant to be exhaustive, we made a complementary validation on a random set of species from our dataset. We used the R function *sample* to randomly draw 100 species from a curated list of 1241 species in our database (this list did not include genomes for which a genus but not a species was defined, such as *Glacieola sp*.). We identified capsule systems in 40 of the 100 species. We then sought to confirm the presence of capsule in the latter (they include 52.5% of free-living, 30% facultative pathogens, 12.5% commensals and 5% of mutualists) by analyzing the primary scientific literature. For those species for which we did not detect a capsule system, we did not seek further validation as negative results are not systematically reported.

### Identification of the core genome of Enterobacteria

We identified the core genome of 131 enterobacterial genomes belonging mostly to *E*. *coli* and *Salmonella spp*., but also *Shigella spp*., *Citrobacter*, *Cronobacter*, *Klebsiella*, and *Enterobacter* (see S8 Table for the complete list of genomes). We followed a previously published methodology [92]. Briefly, orthologs were identified as bidirectional best hits, using end-gap free global alignment, between the proteome of *E*. *coli* K12 MG1655 and each of the 130 other proteomes. We discarded hits with less than 60% similarity in amino acid sequence or more than 20% difference in protein length. The list of orthologs for every pairwise comparison was then curated to take into account the high conservation of gene neighborhood at this phylogenetic scale [93]. We defined positional orthologs as bidirectional best hits adjacent to at least four other pairs of bidirectional best hits within a neighborhood of 10 genes (five genes upstream and five downstream). The core genome was defined as the intersection of pairwise lists of positional orthologs and consisted of 759 gene families.

### Construction of phylogenetic trees

To control for phylogenetic independence of data at the genome-level, we aligned the 16S rRNA using secondary structure models with the program SSU_Align v0.1 [94] of 2440 bacterial genomes. The alignment was trimmed with trimAl v1.4 [95] using the option -*noallgaps* to delete only the gap positions but not the regions that are poorly conserved. The 16S rRNA phylogenetic tree was infered using IQTREE v.1.4.3 [96] under the GTR+I+G4 model with the options–*wbtl* (to conserve all optimal trees and their branch lengths), and–*bb 1000* to run the ultrafast bootstrap option with 1000 replicates. Two hundred and eleven genomes from our database were excluded from the final phylogenetic tree because identical 16S sequences were already present in the multiple alignment. When data was analyzed at the species level, a 16S rRNA gene per species was chosen by the Bash function RANDOM (from all the available genomes of the species) from the secondary structure alignment and a new phylogenetic tree constructed as above.

To build the core-genome phylogenetic tree of the Enterobacteria, we aligned each core gene family at the amino acid level with MAFFT v7.215 (default options) [90], trimmed non-informative positions with BMGE v1.12 (default options and—t AA) [97], and concatenated the alignments. The tree of the concatenate was built using IQTREE v.1.3.10 under the GTR+I+G4 model [96].

In both trees, the model used was the one minimizing the Bayesian Information Criterion (BIC) among all models available (option -m TEST in IQTREE).

### Controls for phylogenetic dependence and genome size

All phylogenetic corrections were done using the 16S rRNA tree of Bacteria. We restricted our phylogenetic controls to Bacteria, because the inclusion of Archaea reduced very much the phylogenetic signal (resulting from a shorter multiple alignment) and clumped together many species’ 16S sequences.

The presence of phylogenetic signal in the evolution of traits was estimated with Pagel’s lambda using the *phylosig* function of the phytools package v.0.5–20 for R [42] and the aforementioned 16S rRNA phylogenetic tree. To estimate the phylogenetic signal across capsule groups, instead of using the 16S rRNA tree, we built new trees comprising only the 16S rRNA sequences of the genomes for which we detected the given capsule groups. To control for the effect of the uncertainty in phylogenetic inference on the key positive results, we produced 1000 bootstrap trees (options -*wbtl* -*bb* 1000 in IQTREE) and randomly selected 100 of those trees. We then ran each key analysis (those in the figures, either *GEE*, *fitPagel* or *phylosig* functions) using the different trees. The distribution of the 100 *P* values of each analysis is presented in S5 Fig.

We tested the significance of the co-occurrence of capsule groups, with the default method (*fitMk*) of the *fitPagel* function from the phytools package (v0.5–52 maps v3.1.0). This function assumes an ARD—all rates different, which allows different rates at all transitions- substitution model for both characters and gives the probability that they are independent (the rates of transitions of each character are independent of the other character).

We controlled the associations between traits for phylogenetic dependence whenever one of their lambda’s *P* values was less than 0.05. We used the *pic* function to make independent contrast analysis of continuous data and the *compar*.*gee* function to analyze associations between discrete and continuous variables using generalized estimation equations (GEE). Both were computed with the functions included in the ape v.3.5 package for R [98]. We also controlled associations for the effect of genome size by fitting linear regression models using *aov* from R.

### Metagenome analyses

We selected from MG-RAST the metagenomes matching at least one species of our complete genome database and obtained from four environmental categories (subclasses indicated in S8 Table): (i) water (fresh, marine and spring water), (ii) soil (agricultural, dessert, forest, tundra and grasslands), (iii) air (indoor, mammal), and (iv) host-associated (human, other mammals, arthropods, aquatic organisms and plant). These categories are broad and heterogeneous (they put together many different environments). They are used to provide a very coarse-grained classification of the type of environment of each species.

We used 16S rRNA assembled reads to identify and quantify the presence of species from the complete genome dataset in the environmental samples. All analyses were performed at the species level rather than at the strain level because 16S rRNA does not allow resolving phylogenetic structure below the species level. For consistency with previous analyses, Archaea were also excluded from the 16S environmental datasets. First, for each metagenome we identified the 16S matching each of the species in our database using BLASTN v 2.2.28 (selected hits with more than 97% sequence identity and with alignments covering at least 90% of the query sequence). The relative abundance of each species was then calculated by dividing the number of 16S rRNA sequences in each metagenome by the total number of sequences. This information was used to draw the frequency of species with capsule systems in each environmental category and subcategory. To validate the analysis, we searched for well-known pathogens and quantified the frequency in which they appeared across metagenomes of each environmental subcategory (S11 Table).

### Other software and packages

Sequence identities and similarities were calculated with needle function (default settings) included in the EMBOSS 6.6 package. Phylogenetic trees were produced with iTol v3.0 [99]. Statistical analysis and graphs were done with R version 3.2.0 and the packages ggplot2 and RColorBrewer, unless stated otherwise. PMCMR [100], stats and NCstats [101] packages for R were used for *post hoc* pairwise multiple comparisons of mean ranks and data manipulation.

### Availability

We have made publicly available the methods to detect capsules. CapsuleFinder can be used locally using the program MacSyFinder [33], freely available for download at https://github.com/gem-pasteur/macsyfinder. We recommend the use of our models without the option "all' (as recommended in the documentation of the program). It can also be queried on a dedicated webserver within the Galaxy platform (https://galaxy.pasteur.fr/root?tool_id=toolshed.pasteur.fr/repos/odoppelt/capsulefinder/CapsuleFinder/1.0.2). The protein profiles and capsule models used in this study are accessible at https://research.pasteur.fr/fr/tool/capsulefinder/. The models are written in a simple XML grammar in plain text files to allow user modifications (see documentation in http://macsyfinder.readthedocs.io/en/latest/). The results of MacSyFinder can be visualized with MacSyView, available online at http://macsyview.web.pasteur.fr. The capsules detected in this study, their genomic localization and organization are collected in an accessible database, CapsuleDB, http://macsydb.web.pasteur.fr/capsuledb/_design/capsuledb/index.html.

## Supporting information

S1 Table List of HMM profiles used in this study. (PDF)

S2 Table Results of the validation of our model. (PDF)

S3 Table List of identified false positives and false negatives. (PDF)

S4 Table Correlation between genome size and capsule complexity. The length of the capsule system (*i*.*e*. number of genes in the system) is used as a proxy for capsule complexity. Genome size was log_10_-transformed before analysis. We used Spearman’s rank association (rho) as a measure of correlation.
(PDF)

S5 Table Genomes with more than five capsule systems detected. (PDF)

S6 Table Number of times each capsule type co-occurs in a genome. The diagonal represents the number of genomes in which the same capsule group co-occurs.
(PDF)

S7 Table Statistics for the dependent evolution between pairs of capsule types. This was first calculated by the analysis of contingency tables of co-occurrence (using χ^**2**^). In complement, for each capsule pair, we made the analysis to account for phylogenetic dependence using the *fitPagel* function. We then computed the likelihood ratio and the corresponding *P*-value for each tree (see Methods).
(PDF)

S8 Table Details of the genomes used to generate the Enterobacteria core genome. (PDF)

S9 Table Stepwise multiple regression. Results of the controls for other variables (Z) when building a linear model where presence or absence of the capsule is the dependent variable (Y) and the focal variable is the independent variable (X). The complete linear model is Y~X+Z. The analysis was done using a stepwise multiple regression (forward using the minimum BIC as stop criterion). *N* indicates sample size. Order (*P* value) indicates the order of entry of the focal variable in the stepwise regression (the *P* value is computed for the Wald χ^**2**^-test). Control (order, BIC) indicates the variables controlled for, their order of entry (ranked by contribution to the linear model), and if the variable is regarded as significant using the BIC test.
(PDF)

S10 Table Summary of metagenomic data. (PDF)

S11 Table Presence of selected pathogens in metagenomes. Numbers and color shading represent the percentage of metagenomes per sub-environment in which each species is present (from white to blue, 0 to 100% respectively).
(PDF)

S1 Fig Cumulative density of the number of genes (system length). The graph shows the cumulative density function of the number of genes of each capsule group. There are significant differences in the number of genes (system length) per capsule group and subgroup as measured by the test: Kruskal-Wallis, *df* = 7, *P* < 0.0001. The *post hoc* Tukey HSD was significant for all pairwise analyses between ABC and Group I capsules against all other groups.
(TIF)

S2 Fig Distribution of distances between two capsule systems of the same group within a replicon. Log-scaled X-axis represents distance in kilobase pairs.
(TIF)

S3 Fig Correlation between capsule systems and life style traits. Cladogram based on the 16S rRNA sequence of species. For species with more than one sequenced genome in our database, the 16S rRNA sequence was randomly chosen. Squares on the outer part of the tree indicate, from inner circle to outer circle, whether species (i) have a capsule system, (ii) whether they are pathogens, (iii) whether they display facultative interactions with the host, (iv) whether they have facultative respiration modes and (v) whether they are motile or not. Empty squares indicate the absence of a trait whereas full squares indicate presence. Absence of squares indicate that data on the trait was not recovered for the species. Branching events with a blue dot highlight bootstrap values below 80. Dot size is proportional to bootstrap value.
(TIF)

S4 Fig Association between the presence of capsules and pathogenic traits. **A**. Frequency of C_sp_+ and C_sp_- in function of their growth class ** *P* < 0.01 for significant dependent evolution between growth class and presence of capsule. **B**. Average infection dose values (ID_50_) expressed in the log scale. **C-D**. Frequency of C_sp_+ and C_sp_- in function of the motility (**C**) and subversion or the ability to escape killing by phagocytes (**D**). Owing to lack of statistical power, association between capsules and ID_50_ (B) and phagocyte killing or subversion (D) were not statistically significant. ID_50_ data was collected for the purpose of a previous study [49]. The data was exclusively measured in human hosts, with one sole exception, the one of *H*. *pylori*. References indicating only upper or lower bounds for ID_50_ were discarded except when they consisted of very high lower limits or very low higher limits (e.g. <30 for *H*. *ducreyi* or >2*10^10^ for *G*. *vaginalis*) in which case the imprecision does not change qualitatively the character of being a very low or very high ID_50_ relative to the other values. ID_50_ values taken from immuno-compromised patients or peculiar uptakes (e.g. oral route with antacids) were excluded. To compensate for the large variance in observed values in some pathotypes, the sources of data on infectious dose were used and the average values, which are the result of arithmetic averages over the log-transformed range values, were calculated. Phagocytosis survival data, was recovered from published evidence on the ability of bacteria to survive and/or replicate in professional phagocytes and/or of being able to kill professional phagocytes. As professional phagocytes, neutrophils, monocytes, macrophages, dendritic cells, and mast cells were considered, although most evidence concerns macrophages and neutrophils. Antigenic variation or the use of specific mechanisms to actively prevent phagocytosis without killing the professional phagocyte are not included in this list. Details concerning motility was taken from the reference book [73].
(TIF)

S5 Fig *P*-value distribution after application of phylogenetic controls. To test for the dependence between presence of capsule and bacterial lifestyle, the *fitPagel* function was performed on 100 trees obtained by bootstrap experiments on the multiple alignment. We plot the distribution of the corresponding *P* values (log-scale) in the graphs. Blue dashed lines indicate the median. To test the association of bacterial doubling time with presence of capsule, we ran *compar*.*gee* function on 100 independent trees. To analyze whether there was phylogenetic inertia in the growth class (fast or slow-growing bacteria), we ran *phylosig* function and Pagel’s lambda is displayed.
(TIF)

S6 Fig Environmental distribution and abundance of species in relation to the presence of capsule system in their genomes. **A**. Relative abundance of C_sp_+ and C_sp_- across environments. Y-axis is in log scale. Statistics reflect significant differences in the relative abundance between C_sp_+ and C_sp_-, non-parametric Wilcoxon test and Benjamini & Hochberg *post hoc* correction ** *P* < 0.01, ***P < 0.0001. **B**. Average relative of abundance of C_sp_+ and C_sp_- across metagenomes in different body locations.
(TIF)

S7 Fig Distribution of bacterial species in our database in function of the type of ecological interaction with hosts (A) and type of host (B). NA indicates that information was lacking or ambiguous.
(TIF)

S1 Dataset Datasets used in this study: (i) list of prokaryotic genomes analysed, (ii) list of capsule systems identified, (iii) bacterial lifestyle, (iv) metagenomes analysed. (XLSX)

S1 Text Detection of false positives and false negatives. (PDF)
