# Supplementary material for: Abundance and co-occurrence of extracellular capsules increase environmental breadth: Implications for the emergence of pathogens
Source: PLoS Pathog. 2017 Jul 24;13(7):e1006525. doi: 10.1371/journal.ppat.1006525 (PMC5542703; doi:10.1371/journal.ppat.1006525)
Supplement: S1 Table — (PDF) [file ppat.1006525.s001.pdf]

**Table S1. List of HMM profiles used in this study.**

| Capsule system | Protein name | HMMer profile name | Other common names | Profile length | # seq. | Description /Function | Source |
|---|---|---|---|---|---|---|---|
| Group I | Wzx | Wzx.hmm | | 274 | 18 | Flippase, polysaccharide biosynthesis protein | PF01943.13 |
| Group I (diderm) | Wzi | Wzi.hmm | | 484 | 54 | Chain length regulator | This study |
| Group I (monoderm) | Wzd | Wzz.hmm | Wzz,CpsC | 152 | 37 | Chain length determinant protein | PF02706.11 |
| | Wze | Wze.hmm | CpsD | 154 | 59 | AAA domain | PF13614.2 |
| | Wzg | Wzg.hmm | CpsA | 152 | 36 | Cell envelope-related transcriptional attenuator domain | PF03816.10 |
| | Wzh | Wzh.hmm | CpsB | 233 | 233 | | This study |
| ABC | KpsM | KpsM.hmm | Wzm | 245 | 199 | ABC-2 transporter | This study |
| | KpsT | KpsT.hmm | Wzt | 212 | 23 | ABC-2 transporter | This study |
| | KpsE | KpsE.hmm | | 354 | 132 | Membrane adaptor protein | This study |
| | KpsC | KpsC.hmm | | 584 | 105 | Involved in capsule export? | This study |
| | KspS | KpsS.hmm | | 389 | 98 | Involved in capsule export? | This study |
| Group IV _s | YshA | YshA.hmm | | 222 | 4 | Oligogalacturonate-specific porin protein (KdgM) | PF06178.9 |
| | YihO | YihO.hmm | YihP | 427 | 90 | MFS/sugar transport protein | PF13347.2 |
| | YihQ | YihQ.hmm | | 723 | 607 | MFS2/sugar transport protein | This study |
| | YihR | YihR.hmm | GalM | 301 | 65 | Aldose 1-epimerase | PF01263.17 |
| | YihS | YihS.hmm | | 346 | 15 | N-acylglucosamine 2-epimerase (GlcNAc 2-epimerase) | PF07221.7 |
| | YihT | YihT.hmm | | 235 | 16 | DeoC/LacD family aldolase | PF01791.5 |
| | YihU | YihU.hmm | | 290 | 1332 | Sulfoacetaldehyde reductase | This study |
| | kinYihV | YihV.hmm | | 302 | 52 | pfkB family carbohydrate kinase | PF00294.20 |
| | repYihW | YihW.hmm | | 253 | 1081 | Carbohydrate sensor | This study |
| Group IV _e | YmcA | YmcA.hmm | YbjH | 659 | 55 | Exopolysaccharide biosynthesis protein | PF06082.7 |
| | YmcB | YmcB.hmm | GfcC | 228 | 9 | Capsule biosynthesis GfcC | PF06251.7 |
| | YmcC | YmcC.hmm | GfcB, YjbF | 203 | 44 | Group 4 capsule polysaccharide lipoprotein GfcB | PF11102.4 |
| | YmcD | YmcD.hmm | | 101 | 71 | | This study |
| | YccZ | YccZ.hmm | GfcE | 306 | 891 | Outer membrane transport protein | This study |
| Group IV _f | | FF1.hmm | | 372 | 4 | | This study |
| | | FF2.hmm | | 256 | 29 | Glycosyltransferase family 8 | PF01501.16 |
| | | FF3.hmm | | 436 | 3 | | This study |
| Syn_CPS3 | Cps3S | Cps3S.hmm | | 446 | 223 | Processive glycosyltranferase, polymerase and translocase | This study |
| | Cps3M | Cps3M.hmm | | 485 | 1193 | Phosphomannomutase | This study |

| | | | | | | | |
|---|---|---|---|---|---|---|---|
| | Cps3D | Cps3D.hmm | | 431 | 1218 | UDP-glucose dehydrogenase | This study |
| | Cps3U | Cps3U.hmm | | 229 | 28 | Glycosyltransferase-like family 2 | PF13641.2 |
| | Cps3C | Cps3C.hmm | | 256 | 1106 | Uridyltransferase | This study |
| Syn_HAS | HasA | HasA_gt_synth.hmm | | 392 | 3 | Processive glycosyltranferase, polymerase and translocase | This study |
| | HasB | HasB.hmm | | 432 | 632 | UDP-glucose dehydrogenase | This study |
| | HasC | HasC.hmm | | 249 | 601 | UDP-glucose pyrophosphorylase | This study |
| PGA | CapA | CapA.hmm | PgsA, YwtB | 257 | 118 | Bacterial capsule synthesis protein | PF09587.6 |
| | CapB | CapB.hmm | PgsB, YwtA | 192 | 56 | Mur ligase middle domain | PF08245.8 |
| | CapC | CapC.hmm | PgsC, YwsC | 119 | 61 | Capsule biosynthesis | PF14102.2 |
| | CapD | CapD.hmm | PgsD | 512 | 43 | Gamma-glutamyltranspeptidase | PF01019.17 |
| | CapE | CapE.hmm | PgsE | 54 | 20 | | This study |
| | PgsS | PgsS.hmm | | 405 | 6 | | This study |
| Shared | Wza | Wza_KpsD.hmm | KpsD,YzzC | 82 | 672 | Polysaccharide biosynthesis/export protein | PF02563.12 |
| | Wzb | Wzb.hmm | Etb | 142 | 15 | Low molecular weight phosphotyrosine protein phosphatase | PF01451.17 |
| | Wzc | Etk.hmm | Etk, ptk | 618 | 1329 | Polysaccharide biosynthesis/export protein, inner membrane | This study |
| | Wzy | Wzy.hmm | | 381 | 34 | Polysaccharide polymerase | This study |
| Sugar-modyfying enzymes | | Glycos_transf_1.hmm | | 172 | 46 | Glycosyl transferases group 1 | PF00534.17 |
| | Ugd | Ugd.hmm | | 188 | 21 | UDP-glucose/GDP-mannose dehydrogenase family, NAD binding domain | F03721.10 |
| | | NTP_Tr.hmm | GalF | 248 | 40 | Nucleotidyl transferase | PF00483.19 |
| | | Man_Deh.hmm | | 332 | 270 | GDP-mannose 4,6 dehydratase | PF16363.1 |
| | | Epimerase.hmm | | 241 | 96 | NAD dependent epimerase/dehydratase family | PF01370.18 |
| | | GDP_Man_Dehyd.hmm | | 332 | 263 | GDP-mannose 4,6 dehydratase | PF16363.2 |
| | | Acetyltransf_1.hmm | | 80 | 222 | Acetyltransferase (GNAT) family | PF00583.22 |
| | | Glu_Tr_2.hmm | | 170 | 146 | Glycosyl transferase family 2 | PF00535.22 |
| | | Gly_Tr.hmm | | 367 | 16 | CDP-Glycerol:Poly(glycerophosphate) glycerophosphotransferase | PF04464.10 |

| | | | | | | |
|---|---|---|---|---|---|---|
| WchA | WchA.hmm | WbaP, CpsE | 153 | 1966 | Bacterial sugar transferase | PF02397.12 |
| | KpsF.hmm | | 307 | 419 | Arabinose-5-phosphate epimerase | This study |
| | KpsU.hmm | | 240 | 483 | CMP-Kdo synthetase | This study |
