# Supplementary material for: Abundance and co-occurrence of extracellular capsules increase environmental breadth: Implications for the emergence of pathogens
Source: PLoS Pathog. 2017 Jul 24;13(7):e1006525. doi: 10.1371/journal.ppat.1006525 (PMC5542703; doi:10.1371/journal.ppat.1006525)
Supplement: S2 Table — (PDF) [file ppat.1006525.s002.pdf]

**Species**	**# genomes**	**% detected capsule**	**Lifestyle**	**Validation**	**Reference**
Psychrobacter arcticus	1	100	Free-living	PhD Thesis, *P. articus* forms capsule in the presence of salt	[1]
Bacillus selenitireducens	1	100	Free-living	Suggested in *B. selenitireducens*	[2]
Methanoregula formicicum	1	100	Free-living	-	-
Lactobacillus acidophilus	3	100	Commensal	Experimental evidence from *L. acidophilus*	[3]
Nostoc punctiforme	1	100	Free-living	Experimental evidence in another *Nostoc* species	[4]
Burkholderia pseudomallei	9	100	Fac. pathogen	Experimental evidence from *B. pseudomallei* for Group I and ABC	[5, 6]
Glaciecola nitratireducens	1	100	Free-living	-	-
Thermosphaera aggregans (Archaea)	1	100	Free-living	-	-
Nitrobacter winogradskyi	1	100	Free-living	Experimentally observed in *N. winogradawsky*	[7]
Lactobacillus buchneri	2	100	Commensal		
Bacillus subtilis	11	91	Free-living		[8]
Teredinibacter turnerae	1	100	Commensal	-	-
Cyclobacterium marinum	1	100	Free-living	Mentioned in a French website on Sphingobacteria taxonomy	[9]
Geobacter lovleyi	1	100	Free-living	Bioinformatically detected in *G. lovleyi*	[10]
Aerococcus urinae	1	100	Fac. pathogen	Another *Aerococcus* species is capsulated	[11]
Methylobacterium radiotolerans	1	100	Fac. mutualist		
Leptospira interrogans	3	100	Fac. pathogen	Bioinformatically detected the genes for PGA production	[12]
Pediococcus claussenii	1	100	Free-living	Pediococcus genus does not produce a capsule according to Bergey's	
Paludibacter propionicigenes	1	100	Free-living	-	-
Nitrobacter hamburgensis	1	100	Free-living	Bioinformatically detected in another *Nitrobacter* species	[13]
Bifidobacterium thermophilum	1	100	Commensal	Experimentally observed in *B. thermophilum*	[14]
Streptococcus iniae	1	100	Fac. pathogen	Experimental evidence from *S. iniae*	[15]
Marinobacter adhaerens	1	100	Fac. mutualist	-	-
Burkholderia ambifaria	2	100	Fac. pathogen	-	-
Burkholderia thailandensis	2	100	Fac. pathogen	Bioinformatically detected in *B. thailandensis*	[16]
Brevundimonas subvibrioides	4	100	Free-living	-	-
Pseudomonas stutzeri	6	100	Fac. pathogen		[17]
Citrobacter koseri	1	100	Fac. pathogen	-	-
Desulfovibrio africanus	1	100	Free-living	Bioinformatically in another Desulfovibrio species	[18]
Erwinia pyrifoliae	2	100	Fac. pathogen	Experimentally validated in *E. pyrifoliae*	[19]
Burkholderia cepacia	1	100	Fac. pathogen	Experimentally validated in *B. cepacia*	[20]
Cytophaga hutchinsonii	1	100	Free-living	Suggested by its slimy appeareance	[21]
Geobacter sulfurreducens	2	100	Free-living	No capsule was observed in standard lab conditions	[22]
Methylocella silvestris	1	100	Free-living	Experimentally observed in *M. sylvestris*	[23]

Brucella suis	4	100	Fac. pathogen	Most are known to lack a capsule, but some do express one.	[24]
Mycobacterium smegmatis	2	100	Commensal	Experimentally observed in *M. smegmatis*	[25]
Acinetobacter calcoaceticus	1	100	Fac. pathogen	Experimentally validated in *A. calcoaceticus*	[26]
Ferroplasma acidarmanus	1	100	Free-living	-	-
Clostridium clariflavum	1	100	Free-living	Observed in another *Clostridium* species	[27]
Nitrosococcus halophilus	1	100	Free-living	-	-

1. Ayala-del-Rio HL, Chain PS, Grzymski JJ, Ponder MA, Ivanova N, Bergholz PW, et al. The genome sequence of Psychrobacter arcticus 273-4, a psychroactive Siberian permafrost bacterium, reveals mechanisms for adaptation to low-temperature growth. Applied and environmental microbiology. 2010;76(7):2304-12. doi: 10.1128/AEM.02101-09. PubMed PMID: 20154119; PubMed Central PMCID: PMC2849256.
2. Blum JS, Bindi AB, Buzzelli J, Stolz JF, Oremland RS. *Bacillus arsenicoselenatis*, sp nov, and *Bacillus selenitireducens*, sp nov: two haloalkaliphiles from Mono Lake, California that respire oxyanions of selenium and arsenic. Arch Microbiol. 1998;171(1):19-30. PubMed PMID: WOS:000077652200004.
3. Lorca G, Torino MI, de Valdez GF, Ljungh A. Lactobacilli express cell surface proteins which mediate binding of immobilized collagen and fibronectin. Fems Microbiol Lett. 2002;206(1):31-7. doi: Doi 10.1111/J.1574-6968.2002.Tb10982.X. PubMed PMID: WOS:000173284600005.
4. Han PP, Sun Y, Jia SR, Zhong C, Tan ZL. Effects of light wavelengths on extracellular and capsular polysaccharide production by *Nostoc flagelliforme*. Carbohyd Polym. 2014;105:145-51. doi: 10.1016/j.carbpol.2014.01.061. PubMed PMID: WOS:000335289400019.
5. Perry MB, Maclean LL, Schollaardt T, Bryan LE, Ho M. Structural characterization of the lipopolysaccharide O-antigens of *Burkholderia pseudomallei*. Infection and immunity. 1995;63(9):3348-52. PubMed PMID: WOS:A1995RQ79200015.
6. Reckseidler-Zenteno SL, Viteri DF, Moore R, Wong E, Tuanyok A, Woods DE. Characterization of the type III capsular polysaccharide produced by Burkholderia pseudomallei. Journal of medical microbiology. 2010;59(12):1403-14. doi: 10.1099/jmm.0022202-0. PubMed PMID: WOS:000285271900002.
7. Szwerinski H, Gaiser S, Bardtke D. Immunofluorescence for the quantitative-determination of nitrifying Bacteria - interference of the test in biofilm reactors. Appl Microbiol Biot. 1985;21(1-2):125-8. PubMed PMID: WOS:A1985AEN4100022.
8. Ashiuchi M, Soda K, Misono H. A poly-gamma-glutamate synthetic system of *Bacillus subtilis* IFO 3336: Gene cloning and biochemical analysis of poly-gamma-glutamate produced by *Escherichia coli* clone cells. Biochem Bioph Res Co. 1999;263(1):6-12. doi: Doi 10.1006/Bbrc.1999.1298. PubMed PMID: WOS:000082712300002.
9. Garcia JL, Cayol JL, Roger P. Taxonomie des Procaryotes.Sphingobacteriia, Classe III des Bacteroidetes: Phylum BXXII du Domaine Bacteria 2007 [updated May 2012; cited 2016]. Available from: http://garciajeanlouis9051.perso.neuf.fr/index.html.
10. Wagner DD, Hug LA, Hatt JK, Spitzmiller MR, Padilla-Crespo E, Ritalahti KM, et al. Genomic determinants of organohalide-respiration in *Geobacter lovleyi*, an unusual member of the Geobacteraceae. Bmc Genomics. 2012;13. doi: Artn 200
10.1186/1471-2164-13-200. PubMed PMID: WOS:000306555100001.

11. Stewart JE, Cornick JW, Zwicker BM, Arie B. Studies on the virulence of *Aerococcus viridans* (var.) homari, the causative agent of gaffkemia, a fatal disease of homarid lobsters. Dis Aquat Organ. 2004;60(2):149-55. doi: Doi 10.3354/Dao060149. PubMed PMID: WOS:000224225800008.
12. Ren SX, Gang F, Jiang XG, Zeng R, Miao YG, Xu H, et al. Unique physiological and pathogenic features of *Leptospira interrogans* revealed by whole-genome sequencing. Nature. 2003;422(6934):888-93. doi: 10.1038/nature01597. PubMed PMID: WOS:000182432600054.
13. Norton JM, Klotz MG, Stein LY, Arp DJ, Bottomley PJ, Chain PS, et al. Complete genome sequence of *Nitrosospira multiformis*, an ammonia-oxidizing bacterium from the soil environment. Applied and environmental microbiology. 2008;74(11):3559-72. doi: 10.1128/AEM.02722-07. PubMed PMID: 18390676; PubMed Central PMCID: PMC2423025.
14. Kudo H, Kimura N, Suzuki M, Cheng KJ, Costerton JW, Mitsuoka T. Electron-microscopic, biochemical and physiological-studies of *Bifidobacterium pseudolongum* Ss-24 and *Bifidobacterium thermophilum* Ss-19. International Journal of Medical Microbiology Virology Parasitology and Infectious Diseases. 1989;271(3):263-71. PubMed PMID: WOS:A1989AU36100001.
15. Lowe BA, Miller JD, Neely MN. Analysis of the polysaccharide capsule of the systemic pathogen *Streptococcus iniae* and its implications in virulence. Infection and immunity. 2007;75(3):1255-64. doi: 10.1128/IAI.01484-06. PubMed PMID: WOS:000244733900020.
16. Sim BMQ, Chantratita N, Ooi WF, Nandi T, Tewhey R, Wuthiekanun V, et al. Genomic acquisition of a capsular polysaccharide virulence cluster by non- pathogenic Burkholderia isolates. Genome Biol. 2010;11(8). doi: Artn R89
10.1186/Gb-2010-11-8-R89. PubMed PMID: WOS:000283777600010.
17. Krotzky A, Werner D. Nitrogen-Fixation in *Pseudomonas stutzeri*. Arch Microbiol. 1987;147(1):48-57. doi: Doi 10.1007/Bf00492904. PubMed PMID: WOS:A1987G077100009.
18. Morais-Silva FO, Rezende AM, Pimentel C, Santos CI, Clemente C, Varela-Raposo A, et al. Genome sequence of the model sulfate reducer *Desulfovibrio gigas*: a comparative analysis within the Desulfovibrio genus. Microbiologyopen. 2014;3(4):513-30. doi: 10.1002/mbo3.184. PubMed PMID: WOS:000347784600003.
19. Kim WS, Schollmeyer M, Nimtz M, Wray V, Geider K. Genetics of biosynthesis and structure of the capsular exopolysaccharide from the Asian pear pathogen *Erwinia pyrifoliae*. Microbiology. 2002;148(Pt 12):4015-24. doi: 10.1099/00221287-148-12-4015. PubMed PMID: 12480905.
20. Chung JW, Altman E, Beveridge TJ, Speert DP. Colonial morphology of Burkholderia cepacia complex genomovar III: implications in exopolysaccharide production, pilus expression, and persistence in the mouse. Infection and immunity. 2003;71(2):904-9. PubMed PMID: 12540572; PubMed Central PMCID: PMC145372.
21. Verma HP, Martin HH. Chemistry and ultrastructure of surface layers in primitive Myxobacteria: *Cytophaga hutchinsonii* and *Sporocytophaga myxococcoides*. Folia Microbiol. 1967;12:248-54. doi: 10.1007/BF02868739.
22. Stukalov O, Korenevsky A, Beveridge TJ, Dutcher JR. Use of atomic force microscopy and transmission electron microscopy for correlative studies of bacterial capsules. Applied and environmental microbiology. 2008;74(17):5457-65. doi: 10.1128/AEM.02075-07. PubMed PMID: WOS:000258829100024.

23. Dunfield PF, Khmelenina VN, Suzina NE, Trotsenko YA, Dedysh SN. *Methylocella silvestris* sp nov., a novel methanotroph isolated from an acidic forest cambisol. Int J Syst Evol Micr. 2003;53:1231-9. doi: 10.1099/ijs.0.02481-0. PubMed PMID: WOS:000185551100002.
24. Huddleson IF. The presence of a capsule on brucella cells. J Amer Vet Med Assoc. 1940;96(708).
25. Sani M, Houben ENG, Geurtsen J, Pierson J, de Punder K, van Zon M, et al. Direct visualization by Cryo-EM of the Mycobacterial capsular layer: A labile structure containing ESX-1-secreted proteins. Plos Pathog. 2010;6(3). doi: ARTN e1000794
10.1371/journal.ppat.1000794. PubMed PMID: WOS:000277720400007.
26. Rosenberg E, Kaplan N, Pines O, Rosenberg M, Gutnick D. Capsular polysaccharides interfere with adherence of *Acinetobacter calcoaceticus* to hydrocarbon. Fems Microbiol Lett. 1983;17(1-3):157-60. PubMed PMID: WOS:A1983QJ69800033.
27. Zhilina TN, Kevbrin VV, Tourova TP, Lysenko AM, Kostrikina NA, Zavarzin GA. Clostridium alkalicellum sp nov, an obligately alkaliphilic cellulolytic bacterium from a soda lake in the Baikal region. Microbiology. 2005;74(5):557-66. doi: Doi 10.1007/S11021-005-0103-Y. PubMed PMID: WOS:000232973900008.
