# Supplementary material for: Abundance and co-occurrence of extracellular capsules increase environmental breadth: Implications for the emergence of pathogens
Source: PLoS Pathog. 2017 Jul 24;13(7):e1006525. doi: 10.1371/journal.ppat.1006525 (PMC5542703; doi:10.1371/journal.ppat.1006525)
Supplement: S3 Table — (PDF) [file ppat.1006525.s003.pdf]

	Capsule group	Genome	Commentary/Explanation
**False positives**	ABC	*Klebsiella pneumoniae* 342 uid59145	We identified two capsule systems (Group I and ABC) in this genome. The ABC-dependent LPS-secretion machinery just downstream of the *bona fide* Group I capsule was misidentified as a capsule system.
	ABC	*Klebsiella pneumoniae* 1084 uid174151	We identified two capsule systems (Group I and ABC) in this genome. The ABC-dependent LPS-secretion machinery just downstream of the *bona fide* Group I capsule was misidentified as a capsule system
	ABC	*Enterobacter aerogenes* EA1509E	We identified two capsule systems (Group I and ABC) in this genome. The ABC-dependent LPS-secretion machinery just downstream of the *bona fide* Group I capsule was misidentified as a capsule system
	Group IV e	*Azoarcus sp*. KH32C	The same capsule system was counted twice: one as Group I and misidentified as Group IV e capsule due to lack of discrimination.
	Group IV e	*Listonella anguillarum* M3	The same capsule system was counted twice: one as Group I and misidentified as Group IV e capsule due to lack of discrimination.
	Syn_CPS3	*Burkholderia vietnamiensis* G4	The same capsule system was counted twice due to the accumulation of glycosyl transferases similar t those present in CPS3 subgroup in a *bona fide* Group I.
	Syn_CPS3	*Chroococcidiopsis thermalis* PCC 7203	The same capsule system was counted twice due to the accumulation of glycosyl transferases similar t those present in CPS3 subgroup in a *bona fide* Group I.
	Syn_CPS3	*Methylibium petroleiphilum* PM1	The same capsule system was counted twice due to the accumulation of glycosyl transferases similar t those present in CPS3 subgroup in a *bona fide* Group I.
	Syn_CPS3	*Parabacteroides distasonis* ATCC 8503	The same capsule system was counted twice due to the accumulation of glycosyl transferases similar t those present in CPS3 subgroup in a *bona fide* Group I.
	Syn capsule	Candidatus *Nitrosopumilus koreensis* AR1 (Archaea)	The same capsule system was accounted twice as Syn_HAS and Syn_CPS3, due to lack of discrimination power.
**False negatives**	Group I	*Bacteroides fragilis* 9343	We identified a full capsule system in the genome but missed six others because the minimum mandatory genes required for a cluster to be complete was not attained.
	Group I	*Bacteroides thetaiotaomicron VPI-5482*	We identified four capsule systems in the genome but missed four others because the minimum mandatory genes required for a cluster to be complete was not attained.
