# Supplementary material for: Abundance and co-occurrence of extracellular capsules increase environmental breadth: Implications for the emergence of pathogens
Source: PLoS Pathog. 2017 Jul 24;13(7):e1006525. doi: 10.1371/journal.ppat.1006525 (PMC5542703; doi:10.1371/journal.ppat.1006525)
Supplement: S4 Table — The length of the capsule system (i.e. number of genes in the system) is used as a proxy for capsule complexity. Genome size was log10-transformed before analysis. We used Spearman’s rank association (rho) as a measure of correlation. (PDF) [file ppat.1006525.s004.pdf]

Before phylogenetic correction:

Capsule group	Average genome size (log10)	Length of capsule systems			Correlation (rho)	*P*-value	Significance
		Min	Max	Median			
Group I	6.67	7	114	19	-0.10	P< 10^{-4}	***
ABC	6.60	5	44	16	-0.09	0.201	n.s.
GroupIV_e	6.70	6	13	7	-0.17	0.195	n.s.
GroupIV_f	6.28	3	4	3	0.26	0.323	n.s.
GroupIV_s	6.69	8	12	10	0.10	0.326	n.s.
Synthase_CPS3	6.57	2	23	4	0.05	0.449	n.s.
Synthase_HAS	6.57	2	4	3	-0.69	0.018	*
PGA	6.53	2	10	3	0.28	0.019	*
Average (all capsules)[a]	6.65			15	0.03	0.17	n.s.
Total (per genome)[b]	6.65			36	0.46	P< 10^{-4}	***

[a] All capsules groups together.
[b] All capsules per genome taken together

After phylogenetic correction:

Capsule group	Pagel's Lambda	Correlation (PIC,rho)	*p*-value (PIC)	GEE[a]	GEE[b]
Group I	0.943	0.033	0.284	P< 10^{-4}	P< 10^{-4}
ABC	0.959	0.112	0.113	P< 10^{-4}	P< 10^{-4}
Synthase_CPS3	0.385	0.079	0.388	P< 10^{-4}	P< 10^{-4}
Synthase_HAS	0.753	-0.517	0.162	0.086	0.482
PGA	0.999	-0.012	0.932	0.252	0.131
Average (all capsules)	0.83	-	-	-	-
Total (per genome)	0.983	0.11	P< 10^{-4}	P< 10^{-4}	P< 10^{-4}

[a] Calculations performed as Capsule length ~ Genome size (log)
[b] Calculations performed as Genome size (log) ~ Capsule length
