# Supplementary material for: Abundance and co-occurrence of extracellular capsules increase environmental breadth: Implications for the emergence of pathogens
Source: PLoS Pathog. 2017 Jul 24;13(7):e1006525. doi: 10.1371/journal.ppat.1006525 (PMC5542703; doi:10.1371/journal.ppat.1006525)
Supplement: S5 Table — (PDF) [file ppat.1006525.s005.pdf]

| Species name | # capsules | Clade | Description | Genome size (bp) | RefSeq Acc |
|---|---|---|---|---|---|
| *Chroococcidiopsis thermalis* PCC 7203 | 8 | Cyanobacteria | Model for dessication tolerance.<br>Adapted to extremely arid, hot and cold deserts. | 6689401 | NC_019695,<br>NC_019696,<br>NC_019699 |
| *Rivularia sp*. PCC 7116 | 8 | Cyanobacteria | Chemoheterotroph from marine water | 8728773 | NC_019678,<br>NC_019679,<br>NC_019686 |
| *Parabacteroides distasonis* ATCC 8503 | 7 | Bacteroidetes | Distal human gut | 4811379 | NC_009615 |
| *Burkholderia xenovorans* LB400 | 7 | Betaproteobacteria | Aerobic polychlorinated biphenyl (PCB) degrader.<br>Isolated from a landfill | 9731138 | NC_007951,<br>NC_007952,<br>NC_007953 |
| *Geitlerinema sp*. PCC 7407 | 6 | Cyanobacteria | Free living chemoheterotroph. | 4681111 | NC_019703 |
| *Hyphomicrobium sp*. MC1 | 6 | Alphaproteobacteria | Aerobic methylotroph, isolated from industrial sewage. | 9515056 | NC_015717 |
| *Burkholderia phenoliruptrix* BR3459a | 6 | Betaproteobacteria | Plant symbiont.<br>Isolated from nodules of *Mimosa flocculosa* | 7651131 | NC_018695,<br>NC_018672,<br>NC_018696 |
| *Granulicella tundricola* | 6 | Acidobacteria | Heterotroph, isolated from tundra soil. Adapted to low temperatures and nutrient-limiting conditions. | 5503984 | NC_015064,<br>NC_015057,<br>NC_015058,<br>NC_015059,<br>NC_015060,<br>NC_015065 |
| *Koribacter versatilis* Ellin345 | 5 | Acidobacteria | Aerobic heterotroph, isolated from an Australian pasture | 5650368 | NC_008009 |
| *Terriglobus roseus* | 5 | Acidobacteria | Aerobic, chemo-organotrophic | 5227858 | NC_018014 |
| *Granulicella mallensis* MP5ACTX8 | 5 | Acidobacteria | Heterotroph, isolated from tundra soil. Adapted to low temperatures and nutrient-limiting conditions. | 6237577 | NC_016631 |
| *Cyclobacterium marinum* DSM 745 | 5 | Bacteoridetes | | 6221273 | NC_015914 |
| *Rhizobium etli* CFN 42<br>*R. etli* bv mimosae Mim1 | 5 | Alphaproteobacteria | Nitrogen-fixing plant symbiont, commonly found in nodules | 6530228<br>7197998 | NC_004041,<br>NC_021905, |

| | | | | | |
|---|---|---|---|---|---|
| | | | | | NC_021906, NC_021907, NC_021908, NC_021909, NC_021910, NC_021911 |
| *Mesorhizobium sp*. BNC1 *Chelativorans sp*. BNC1 | 5 | Alphaproteobacteria | Isolated from industrial sewage, degrades EDTA | 4935185 | NC_008254, NC_008242, NC_008243, NC_008244 |
| *Sinorhizobium medicae* WSM419 | 5 | Alphaproteobacteria | Nitrogen-fixing plant symbiont, commonly found in nodules of *Medicago polymorpha* | 6817576 | NC_009636, NC_009620, NC_009621, NC_009622 |
| *Sinorhizobium meliloti* 1021, AK83, BL225C, SM11, Rm41, GR4, 2011 | 5 | Alphaproteobacteria | Nitrogen-fixing plant symbiont, commonly found in nodules of *Medicago sp*. | 6.6Mb-7.15 Mb | NC_003037, NC_003047, NC_003078, NC_015590, NC_015591, NC_015592, NC_015596, NC_015597, NC_017322, NC_017323, NC_017324, NC_017325, NC_017326, NC_017327, NC_018682, NC_018683, NC_018700, NC_018701, NC_019845 |
| *Azoarcus sp*. KH32C | 5 | Betaproteobacteria | Nitrogen-fixing bacteria, isolated from agricultural soil | 5818755 | NC_020516, NC_020548 |
| *Burkholderia sp*. 383 *Burkholderia lata* 383 | 5 | Betaproteobacteria | Isolated from forest soil in Trinidad | 8676277 | NC_007510, NC_007511, NC_007509 |

| | | | | | |
|---|---|---|---|---|---|
| *Burkholderia ambifaria* MC40-6, AMMD | 5 | Betaproteobacteria | Associated to the maize rhizosphere, a species in the *Burkholderia cepacia* complex | 7.5-7.6 | NC_010551, NC_010552, NC_010557, NC_010553, NC_008390, NC_008391, NC_008392, NC_008385 |
| *Burkholderia sp*. CCGE1001, CCGE1003 | 5 | Betaproteobacteria | Motile, free-living and rhizosphere-colonizing bacterium | 6.8-7.04 | NC_014540, NC_014539, NC_015136, NC_015137 |
| *Burkholderia cenocepacia* MC0-3 | 5 | Betaproteobacteria | Associated to maize roots, isolated in Michigan, USA | 7971389 | NC_010508, NC_010512, NC_010515 |
| *Burkholderia gladioli* BSR3 | 5 | Betaproteobacteria | Isolated from a diseased rice sheath in South Korea | 9052299 | NC_015381, NC_015376, NC_015377, NC_015378, NC_015382, NC_015383 |
| *Burkholderia phymatum* STM815 | 5 | Betaproteobacteria | Efficient nitrogen-fixing symbiont from plant of genus *Mimosa*, isolated from root nodule in French Guiana | 8676562 | NC_010622, NC_010623, NC_010627, NC_010625 |
| *Burkholderia phytofirmans* PsJN | 5 | Betaproteobacteria | Plant-associated endophyte | 10714659 | NC_010681, NC_010676, NC_010679 |
| *Burkholderia vietnamiensis* G4 | 5 | Betaproteobacteria | Isolated from a holding pond at an industrial waste treatment facility, a species in the *Burkholderia cepacia* complex | 8391070 | NC_009256, NC_009255, NC_009254, NC_009230, NC_009229, NC_009228, NC_009227, NC_009226 |
