# Supplementary material for: Abundance and co-occurrence of extracellular capsules increase environmental breadth: Implications for the emergence of pathogens
Source: PLoS Pathog. 2017 Jul 24;13(7):e1006525. doi: 10.1371/journal.ppat.1006525 (PMC5542703; doi:10.1371/journal.ppat.1006525)
Supplement: S6 Table — The diagonal represents the number of genomes in which the same capsule group co-occurs. (PDF) [file ppat.1006525.s006.pdf]

	GroupI	ABC	GroupIV_e	GroupIV_f	GroupIV_s	Syn_CPS3	Syn_HAS	PGA
GroupI	293							
ABC	126	1						
GroupIV_e	56	5	0					
GroupIV_f	0	2	0	0				
GroupIV_s	89	19	31	0	0			
Syn_CPS3	31	2	0	0	0	30		
Syn_HAS	2	0	0	0	0	3	0	
PGA	13	1	0	16	0	6	4	5
