# Supplementary material for: Abundance and co-occurrence of extracellular capsules increase environmental breadth: Implications for the emergence of pathogens
Source: PLoS Pathog. 2017 Jul 24;13(7):e1006525. doi: 10.1371/journal.ppat.1006525 (PMC5542703; doi:10.1371/journal.ppat.1006525)
Supplement: S7 Table — This was first calculated by the analysis of contingency tables of co-occurrence (using χ2). In complement, for each capsule pair, we made the analysis to account for phylogenetic dependence using the fitPagel function. We then computed the likelihood ratio and the corresponding P-value for each tree (see Methods). (PDF) [file ppat.1006525.s007.pdf]

| | | Independent of phylogeny | | Accounting for phylogeny[1] | | | |
|---|---|---|---|---|---|---|---|
| **Capsule 1** | **Capsule 2** | **$X^2$** | ***P*-value $X^2$** | **Independent model likelihood** | **Dependent model likelihood** | **Likelihood ratio** | ***P*-value** |
| ABC | GroupIV_e | 0 | 1 | -988.98 | -988.98 | 0 | 1 |
| ABC | GroupIV_f | 0.02 | 0.88 | -514.43 | -641.54 | -254.22 | 1 |
| ABC | GroupIV_s | 18.34 | < 0.001 | -615.97 | -594.87 | 42.22 | **< 0.001** |
| ABC | PGA | 2.48 | 0.11 | -680.84 | -679.13 | 3.41 | 0.49 |
| ABC | Syn_CPS3 | 6.53 | 0.01 | -802.7 | -798.55 | 8.01 | 0.09 |
| ABC | Syn_HAS | 0.15 | 0.70 | -550.46 | -549.76 | 1.4 | 0.84 |
| ABC | Group I | 32 .86 | < 0.001 | -1491.57 | -1456.55 | 70.03 | **< 0.001** |
| GroupIV_e | GroupIV_s | 415.96 | < 0.001 | -336.245 | -342.73 | 35.93 | **< 0.001** |
| GroupIV_e | PGA | 0.52 | 0.46 | -320.73 | -320.4 | 0.65 | 0.96 |
| GroupIV_e | Syn_CPS3 | 2.01 | 0.15 | -486.56 | -485.84 | 1.43 | 0.84 |
| GroupIV_e | Syn_HAS | 0 | 1 | -190.35 | -190.2 | 0.29 | 0.99 |
| GroupIV_e | Group I | 69.87 | < 0.001 | -1076.19 | -1067.59 | 17.42 | 0.002 |
| GroupIV_e | GroupIV_f | 0 | 1 | -154.32 | -154.22 | 0.22 | 0.99 |
| GroupIV_f | GroupIV_s | 0.01 | 0.90 | -161.49 | -161.41 | 0.15 | 0.99 |
| GroupIV_f | PGA | 550.26 | < 0.001 | -200.85 | -197.48 | 9.81 | **0.04** |
| GroupIV_f | Syn_CPS3 | 0.11 | 0.73 | -370.99 | -370.79 | 0.4 | 0.98 |
| GroupIV_f | Syn_HAS | 0 | 1 | -74.78 | -74.67 | 0.22 | 0.99 |
| GroupIV_f | Group I | 10.26 | < 0.001 | -1030.99 | -1030.79 | 0.42 | 0.98 |
| GroupIV_s | PGA | 1.3 | 0.25 | -327.9 | -327.33 | 1.13 | 0.88 |
| GroupIV_s | Syn_CPS3 | 3.84 | 0.05 | -493.72 | -492.84 | 1.77 | 0.79 |
| GroupIV_s | Syn_HAS | 0 | 1 | -197.51 | -197.27 | 0.48 | 0.98 |
| GroupIV_s | Group I | 118.8 | < 0.001 | -1069.3 | -1064.98 | 10.33 | **0.035** |
| PGA | Syn_CPS3 | 2.72 | 0.1 | -537.4 | -658.42 | -242.04 | 1 |
| PGA | Syn_HAS | 46.96 | < 0.001 | -302.53 | -303.98 | -2.89 | 1 |
| PGA | Group I | 8.52 | 0.003 | -1783.49 | -1783.49 | 0 | 0.99 |
| Syn_CPS3 | Syn_HAS | 2.15 | 0. 14 | -407.02 | -475.42 | -136.81 | 1 |
| Syn_CPS3 | Group I | 14.26 | < 0.001 | -1301.55 | -294.6 | 13.46 | **0.009** |
| Syn_HAS | Group I | 1.27 | 0.26 | -1 | -1033.79 | -27.85 | **1** |

[1]Capsule pairs for which there is no evidence for dependent evolution (using $\chi^2$), the analysis was performed three times, and the median values for likelihoods and *P*-values are shown.
For the capsule pairs for which there was evidence of dependent evolution, we generated 100 bootstrap trees and calculated independently on each tree the likelihoods for the pairs to evolve under the independent and dependent model of evolution. The median values of the 100 likelihoods and *P*-values are shown. (see Methods).
