# Supplementary material for: Abundance and co-occurrence of extracellular capsules increase environmental breadth: Implications for the emergence of pathogens
Source: PLoS Pathog. 2017 Jul 24;13(7):e1006525. doi: 10.1371/journal.ppat.1006525 (PMC5542703; doi:10.1371/journal.ppat.1006525)
Supplement: S8 Table — (PDF) [file ppat.1006525.s008.pdf]

GenBank ID	Species	Strain	Pathotype[1]	Genome size (bp)
NC_009792	*Citrobacter koseri*	ATCC BAA-895		4720462
NC_013716	*Citrobacter rodentium*	ICC168		5346659
NC_017933	*Cronobacter sakazakii*	ES15		4268675
NC_020260	*Cronobacter sakazakii*	Sp291		4344092
NC_013282	*Cronobacter turicensis*	z3032		4384463
NC_015663	*Enterobacter aerogenes*	KCTC 2190		5280350
NC_020181	*Enterobacter aerogenes*	EA1509E		5419609
NC_014121	*Enterobacter cloacae*	subsp. cloacae ATCC 13047		5314581
NC_014618	*Enterobacter cloacae*	SCF1		4814049
NC_016514	*Enterobacter cloacae*	EcWSU1		4734438
NC_018079	*Enterobacter cloacae*	subsp. dissolvens SDM		4968248
NC_018405	*Enterobacter cloacae*	subsp. cloacae ENHKU01		4726582
NC_009778	*Cronobacter sakazakii*	ATCC BAA-894		4368373
NC_000913	*Escherichia coli*	K-12 substr. MG1655	Commensal	4639675
NC_002695	*Escherichia coli*	O157:H7 Sakai	EHEC	5498450
NC_002655	*Escherichia coli*	O157:H7 EDL933	EHEC	5528445
NC_004431	*Escherichia coli*	CFT073	ExPEC-UPEC	5231428
NC_011750	*Escherichia coli*	IAI39	ExPEC-UPEC	5132068
NC_007779	*Escherichia coli*	K-12 substr. W3110	Commensal	4646332
NC_007946	*Escherichia coli*	UTI89	ExPEC-UPEC	5065741
NC_008253	*Escherichia coli*	536	ExPEC-UPEC	4938920
NC_008563	*Escherichia coli*	APEC O1	ExPEC-APEC	5082025
NC_011751	*Escherichia coli*	UMN026	ExPEC-UPEC	5202090
NC_011748	*Escherichia coli*	55989	EAEC	5154862
NC_011745	*Escherichia coli*	ED1a	Commensal	5209548
NC_011742	*Escherichia coli*	S88	ExPEC-UPEC	5032268
NC_011741	*Escherichia coli*	IAI1	Commensal	4700560
NC_011353	*Escherichia coli*	O157:H7 EC4115	EHEC	5572075
NC_009800	*Escherichia coli*	HS	Commensal	4643538
NC_009801	*Escherichia coli*	E24377A	ETEC	4979619
NC_010473	*Escherichia coli*	K-12 substr. DH10B	Commensal	4686137
NC_010498	*Escherichia coli*	SMS-35	Commensal	5068389
NC_011601	*Escherichia coli*	O127:H6 E2348/69	EPEC	4965553
NC_010468	*Escherichia coli*	ATCC 8739	Commensal	4746218
NC_011415	*Escherichia coli*	SE11	Commensal	4887515
NC_012759	*Escherichia coli*	BW2952	Commensal	4578159
NC_012967	*Escherichia coli*	B REL606	Commensal	4629812
NC_012892	*Escherichia coli*	BL21	Commensal	4558947
NC_013361	*Escherichia coli*	O26:H11 11368	EHEC	5697240
NC_013008	*Escherichia coli*	O157:H7 TW14359	EHEC	5528136
NC_013364	*Escherichia coli*	O111:H- 11128	EHEC	5371077
NC_013353	*Escherichia coli*	O103:H2 12009	EHEC	5449314
NC_017626	*Escherichia coli*	O42	EAEC	5241977
NC_013941	*Escherichia coli*	O55:H7 CB9615	EPEC	5386352
NC_017641	*Escherichia coli*	UMNK88	ETEC	5186416

NC_013654	*Escherichia coli*	SE15	Commensal	4717338
NC_017631	*Escherichia coli*	ABU 83972	ExPEC-ABU	5131397
NC_017634	*Escherichia coli*	O83:H1 NRG 857C	AIEC	4747819
NC_017628	*Escherichia coli*	IHE3034	ExPEC	5108383
NC_017632	*Escherichia coli*	UM146	AIEC	4993013
NC_011993	*Escherichia coli*	LF82	AIEC	4773108
NC_017633	*Escherichia coli*	H10407	ETEC	5153435
NC_012971	*Escherichia coli*	BL21	Commensal	4558953
NC_017625	*Escherichia coli*	DH1	Commensal	4630707
NC_017635	*Escherichia coli*	W uid162011	Environmental ?	4900968
NC_017638	*Escherichia coli*	K12 DH1 uid162051	Commensal	4621430
NC_017644	*Escherichia coli*	NA114	ExPEC-UPEC	4971461
NC_016902	*Escherichia coli*	KO11FL	Commensal	4920168
NC_017656	*Escherichia coli*	O55:H7 RM12579	EPEC	5263980
NC_017646	*Escherichia coli*	O7:K1 CE10	ExPEC	5313531
NC_017663	*Escherichia coli*	P12b	-	4935294
NC_017664	*Escherichia coli*	W	Commensal	4897452
NC_017906	*Escherichia coli*	Xuzhou21	EHEC	5386223
NC_017652	*Escherichia coli*	clone D i14 uid162049	ExPEC-UPEC	5038386
NC_017651	*Escherichia coli*	clone D i2 uid162047	ExPEC-UPEC	5038386
NC_017660	*Escherichia coli*	KO11FL	Commensal	5021812
NC_018650	*Escherichia coli*	O104:H4 2009EL-2050	EHEC	5253138
NC_020163	*Escherichia coli*	APEC O78	ETEC	4798435
NC_022364	*Escherichia coli*	LY180	Commensal	4835601
NC_022370	*Escherichia coli*	PMV-1 main	ExPEC	4984940
NC_022648	*Escherichia coli*	JJ1886	ExPEC-UPEC	5129938
NC_016612	*Klebsiella oxytoca*	KCTC 1686		5974109
NC_018106	*Klebsiella oxytoca*	E718		6097032
NC_011283	*Klebsiella pneumoniae*	342		5641239
NC_009648	*Klebsiella pneumoniae*	subsp. pneumoniae MGH 78578		5315120
NC_012731	*Klebsiella pneumoniae*	NTUH-K2044		5248520
NC_016845	*Klebsiella pneumoniae*	subsp. pneumoniae HS11286		5333942
NC_017540	*Klebsiella pneumoniae*	KCTC 2242		5259571
NC_018522	*Klebsiella pneumoniae*	subsp. pneumoniae 1084		5386705
NC_022566	*Klebsiella pneumoniae*	CG43		5166857
NC_022082	*Klebsiella pneumoniae*	JM45		5273813
NC_021232	*Klebsiella pneumoniae*	subsp. rhinoscleromatis strain SB3432		5270770
NC_013850	*Klebsiella variicola*	At-22		5458505
NC_003197	*Salmonella enterica*	subsp. enterica serovar Typhimurium LT2		4857432
NC_003198	*Salmonella enterica*	subsp. enterica serovar Typhi CT18		4809037
NC_004631	*Salmonella enterica*	subsp. enterica serovar Typhi Ty2		4791961
NC_006905	*Salmonella enterica*	subsp. enterica serovar Choleraesuis SC-B67		4755700
NC_010102	*Salmonella enterica*	subsp. enterica serovar Paratyphi B SPB7		4858887
NC_011080	*Salmonella enterica*	subsp. enterica serovar Newport SL254		4827641

NC_011083	*Salmonella enterica*	subsp. enterica serovar Heidelberg SL476	4888768
NC_011094	*Salmonella enterica*	subsp. enterica serovar Schwarzengrund CVM19633	4709075
NC_011149	*Salmonella enterica*	subsp. enterica serovar Agona SL483	4798660
NC_011147	*Salmonella enterica*	subsp. enterica serovar Paratyphi A AKU_12601	4581797
NC_011205	*Salmonella enterica*	subsp. enterica serovar Dublin CT_02021853	4842908
NC_011274	*Salmonella enterica*	subsp. enterica serovar Gallinarum 287/91	4658697
NC_011294	*Salmonella enterica*	subsp. enterica serovar Enteritidis P125109	4685848
NC_006511	*Salmonella enterica*	subsp. enterica serovar Paratyphi A ATCC 9150	4585229
NC_012125	*Salmonella enterica*	subsp. enterica serovar Paratyphi C RKS4594	4833080
NC_016854	*Salmonella enterica*	subsp. enterica serovar Typhimurium D23580	4879400
NC_016810	*Salmonella enterica*	subsp. enterica serovar Typhimurium SL1344	4878012
NC_016860	*Salmonella enterica*	subsp. enterica serovar Typhimurium T000240	4954814
NC_016856	*Salmonella enterica*	subsp. enterica serovar Typhimurium 14028S	4870265
NC_016857	*Salmonella enterica*	subsp. enterica serovar Typhimurium ST4/74	4878013
NC_016831	*Salmonella enterica*	subsp. enterica serovar Gallinarum/pullorum RKS5078	4637962
NC_017623	*Salmonella enterica*	subsp. enterica serovar Heidelberg B182	4750465
NC_016832	*Salmonella enterica*	subsp. enterica serovar Typhi P-stx-12	4768352
NC_017046	*Salmonella enterica*	subsp. enterica serovar Typhimurium 798	4876219
NC_016863	*Salmonella enterica*	subsp. enterica serovar Typhimurium UK-1	4817868
NC_021812	*Salmonella enterica*	subsp. enterica Serovar Heidelberg CFSAN002069	4783952
NC_021814	*Salmonella enterica*	subsp. enterica serovar Typhimurium var. 5- CFSAN001921	4859931
NC_021820	*Salmonella enterica*	subsp. enterica serovar Typhimurium 08-1736	4822189
NC_021844	*Salmonella enterica*	subsp. enterica serovar Bareilly CFSAN000189	4730612
NC_022241	*Salmonella enterica*	subsp. enterica serovar Bovismorbificans 3114	4677483
NC_022221	*Salmonella enterica*	subsp. enterica serovar Gallinarum/pullorum CDC1983-67	4623089
NC_021810	*Salmonella enterica*	subsp. enterica serovar Heidelberg 41578	4793479
NC_020307	*Salmonella enterica*	subsp. enterica serovar Javiana CFSAN001992	4634161
NC_021902	*Salmonella enterica*	subsp. enterica serovar Newport USMARC-S3124.1	4915960
NC_021984	*Salmonella enterica*	subsp. enterica serovar Pullorum S06004	4682599

NC_022525	*Salmonella enterica*	subsp. enterica serovar Thompson RM6836	4707648
NC_021176	*Salmonella enterica*	subsp. enterica serovar Typhi Ty21a	4791958
NC_022544	*Salmonella enterica*	subsp. enterica serovar Typhimurium DT2	4814801
NC_021151	*Salmonella enterica*	subsp. enterica serovar Typhimurium U288	4852606
NC_007613	*Shigella boydii*	Sb227	4519823
NC_010658	*Shigella boydii*	CDC 3083-94	4615997
NC_007606	*Shigella dysenteriae*	Sd197	4369232
NC_004337	*Shigella flexneri*	2a str. 301	4607202
NC_004741	*Shigella flexneri*	2a str. 2457T	4599354
NC_008258	*Shigella flexneri*	5 str. 8401	4574284
NC_017328	*Shigella flexneri*	2002017	4650856
NC_007384	*Shigella sonnei*	Ss046	4825265
NC_016822	*Shigella sonnei*	53G	4988504

[1]ABU asymptomatic bacteriuria; AIEC Adherent invasive; EAEC Enteroaggregative; APEC Avian pathogenic; ExPEC extraintestinal; EHEC Enterohemorrhagic; EPEC enteropathogenic; ETEC enterotoxigenic; UPEC uropathogenic.
