# Supplementary material for: Abundance and co-occurrence of extracellular capsules increase environmental breadth: Implications for the emergence of pathogens
Source: PLoS Pathog. 2017 Jul 24;13(7):e1006525. doi: 10.1371/journal.ppat.1006525 (PMC5542703; doi:10.1371/journal.ppat.1006525)
Supplement: S9 Table — Results of the controls for other variables (Z) when building a linear model where presence or absence of the capsule is the dependent variable (Y) and the focal variable is the independent variable (X). The complete linear model is Y~X+Z. The analysis was done using a stepwise multiple regression (forward using the minimum BIC as stop criterion). N indicates sample size. Order (P value) indicates the order of entry of the focal variable in the stepwise regression (the P value is computed for the Wald χ2-test). Control (order, BIC) indicates the variables controlled for, their order of entry (ranked by contribution to the linear model), and if the variable is regarded as significant using the BIC test. (PDF) [file ppat.1006525.s009.pdf]

**Focal variable (X)**	***N***	**Order (BIC, p-value)**	**Control (Z) (order, BIC)**
Lifestyle	1156	2 (yes, *P*<0.0001)	Genome size (1, yes)
Obligatory/facultative pathogen	336	2 (yes, *P*<0.0001)	Genome size (1, yes)
Minimal doubling time	189	2 (yes, *P*<0.001)	Genome size (1, yes)
Minimal doubling time	189	1 (yes, *P*<0.0001)	Pathogen/Not pathogen (2, yes)
