# Supplementary material for: Abundance and co-occurrence of extracellular capsules increase environmental breadth: Implications for the emergence of pathogens
Source: PLoS Pathog. 2017 Jul 24;13(7):e1006525. doi: 10.1371/journal.ppat.1006525 (PMC5542703; doi:10.1371/journal.ppat.1006525)
Supplement: S10 Table — (PDF) [file ppat.1006525.s010.pdf]

Environment	Subenvironment	# of Metagenomes	# of identified Species
Air	Indoor	8	489
Host	Animal	2015	907
Host	Aquatic organisms	3	17
Host	Arthropod	2	2
Host	Human	2631	935
Host	Mammal	183	304
Host	Mixed	10	149
Host	Other	15	131
Host	Plant	170	578
Host	U	295	798
Soil	Agricultural	9	311
Soil	Desert	41	507
Soil	Forest	600	622
Soil	Grasslands	449	731
Soil	Host-associated	2	121
Soil	Tundra	35	212
Soil	U	178	901
Water	Freshwater	52	514
Water	House-associated	2	728
Water	Marine	1	5
Water	Spring	29	475
Water	U	2	265

U for undefined
