# Supplementary material for: Abundance and co-occurrence of extracellular capsules increase environmental breadth: Implications for the emergence of pathogens
Source: PLoS Pathog. 2017 Jul 24;13(7):e1006525. doi: 10.1371/journal.ppat.1006525 (PMC5542703; doi:10.1371/journal.ppat.1006525)
Supplement: S11 Table — Numbers and color shading represent the percentage of metagenomes per sub-environment in which each species is present (from white to blue, 0 to 100% respectively). (PDF) [file ppat.1006525.s011.pdf]

Environment	Sub-environment	# of Metagenomes	*Acinetobacter baumannii*	*Clostridium difficile*	*Escherichia coli*	*Klebsiella pneumoniae*	*Neisseria meningitidis*	*Pseudomonas aeruginosa*	*Streptococcus pneumoniae*
Air	Indoor	8	13	13	13		25	25	25
Host	Animal	2015	19	12	1		3	13	5
Host	Aquatic organisms	3							
Host	Arthropod	2	50						
Host	Human	2631	22	36	4	9	39	10	35
Host	Mammal	183	10	49		6	1		1
Host	Mixed	10	50						
Host	Other	15	7	40		13	20		
Host	Plant	170	31	34	1	5	8	2	20
Host	U	295	42	41	7	3	2	27	6
Soil	Agricultural	9		33				0	
Soil	Desert	41	7				2	5	
Soil	Forest	600	11	6		1	2	11	1
Soil	Grasslands	449	11	2				2	
Soil	Host-associated	2	50						
Soil	Tundra	35	20	17			3		
Soil	U	178	21	39	3	17	4	7	12
Water	Freshwater	52	17				2	2	6
Water	House-associated	2	100	50		100	100	100	50
Water	Marine	1							
Water	Spring	29	69	3		10	14	3	
Water	U	2	50				50	50	50

U for undefined
