# Supplementary figures and images for: Abundance and co-occurrence of extracellular capsules increase environmental breadth: Implications for the emergence of pathogens
Source: PLoS Pathog. 2017 Jul 24;13(7):e1006525. doi: 10.1371/journal.ppat.1006525 (PMC5542703; doi:10.1371/journal.ppat.1006525)

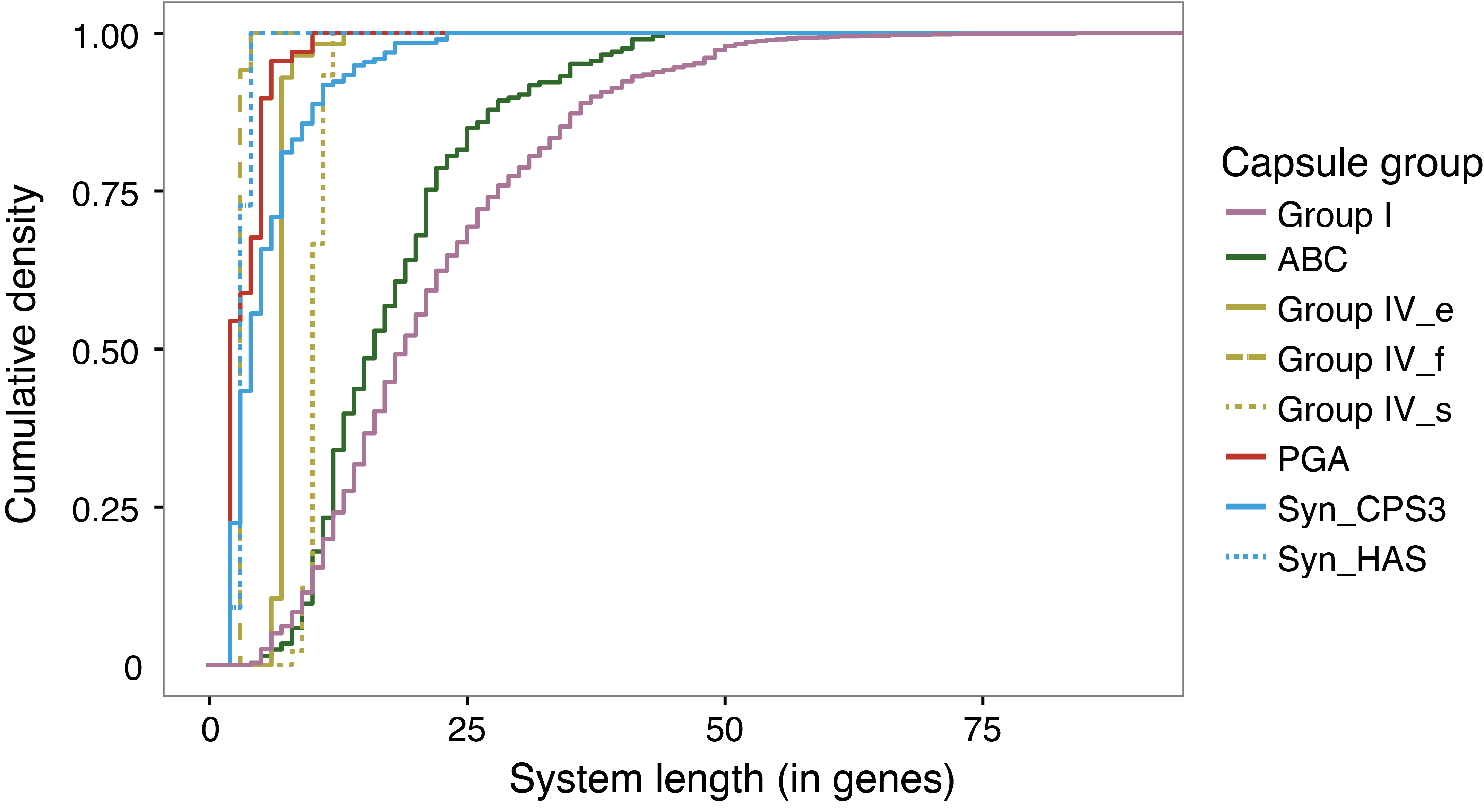

Supplement: S1 Fig — The graph shows the cumulative density function of the number of genes of each capsule group. There are significant differences in the number of genes (system length) per capsule group and subgroup as measured by the test: Kruskal-Wallis, df = 7, P < 0.0001. The post hoc Tukey HSD was significant for all pairwise analyses between ABC and Group I capsules against all other groups. (TIF) [file ppat.1006525.s012.tif]

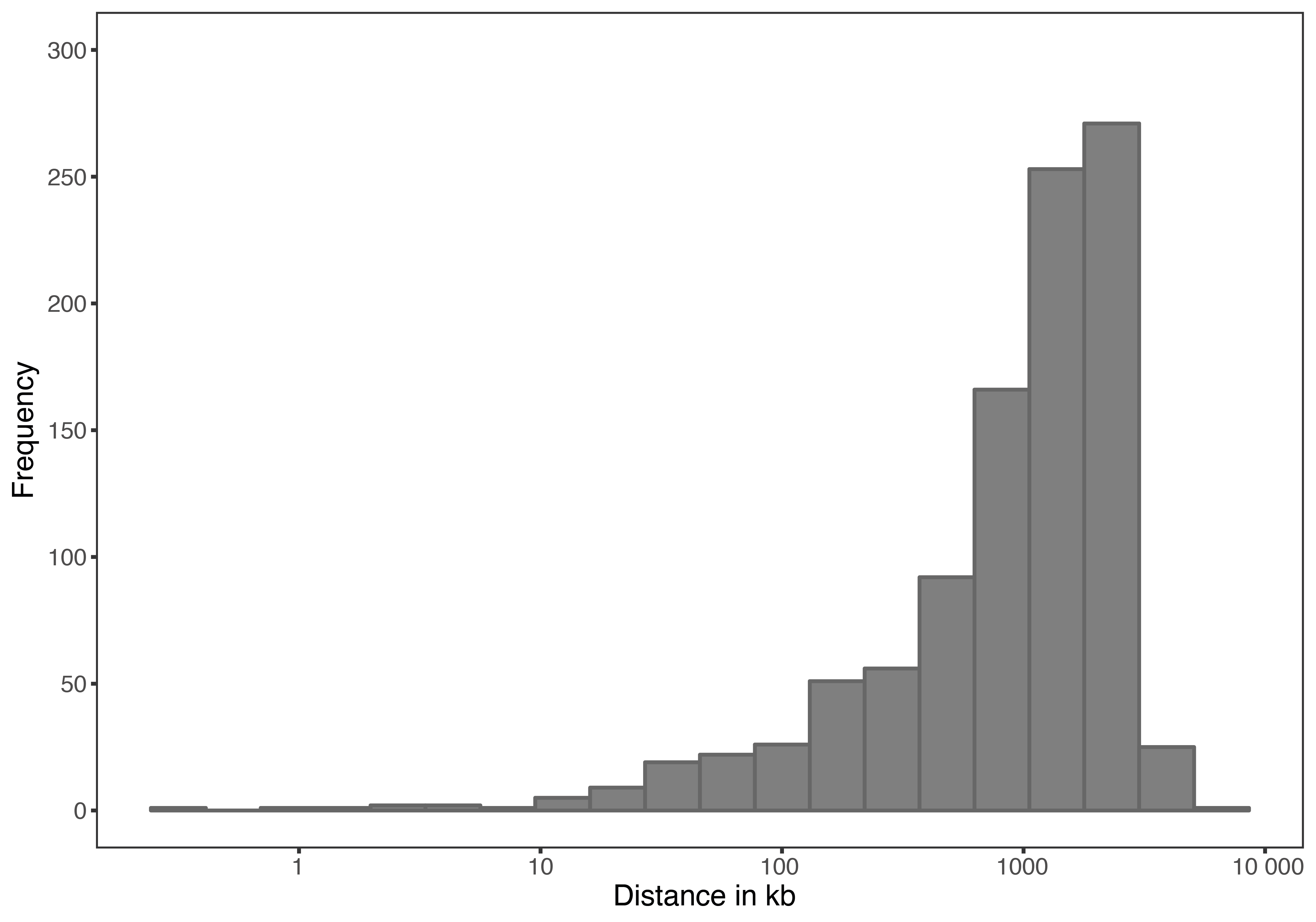

Supplement: S2 Fig — Log-scaled X-axis represents distance in kilobase pairs. (TIF) [file ppat.1006525.s013.tif]

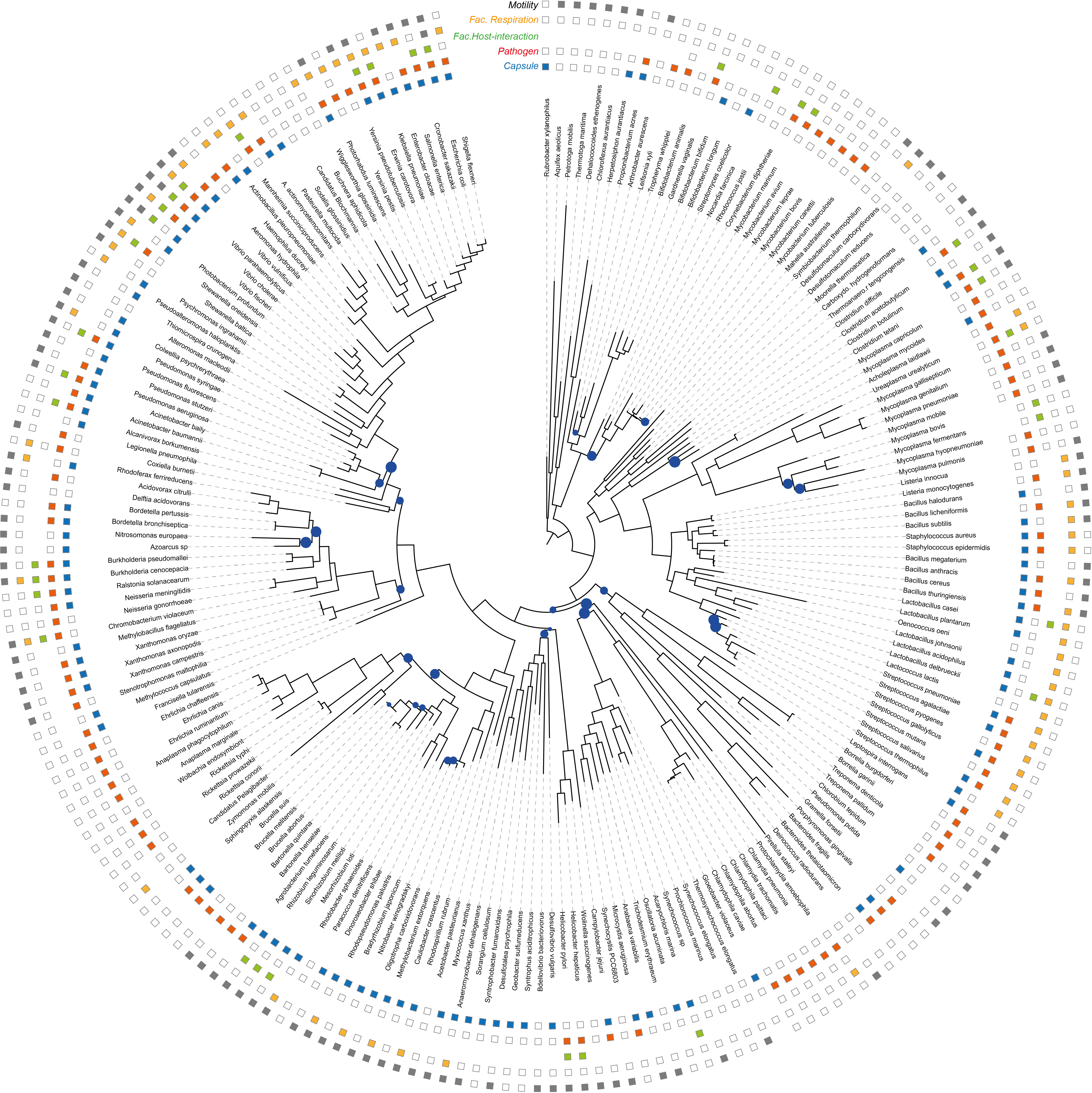

Supplement: S3 Fig — Cladogram based on the 16S rRNA sequence of species. For species with more than one sequenced genome in our database, the 16S rRNA sequence was randomly chosen. Squares on the outer part of the tree indicate, from inner circle to outer circle, whether species (i) have a capsule system, (ii) whether they are pathogens, (iii) whether they display facultative interactions with the host, (iv) whether they have facultative respiration modes and (v) whether they are motile or not. Empty squares indicate the absence of a trait whereas full squares indicate presence. Absence of squares indicate that data on the trait was not recovered for the species. Branching events with a blue dot highlight bootstrap values below 80. Dot size is proportional to bootstrap value. (TIF) [file ppat.1006525.s014.tif]

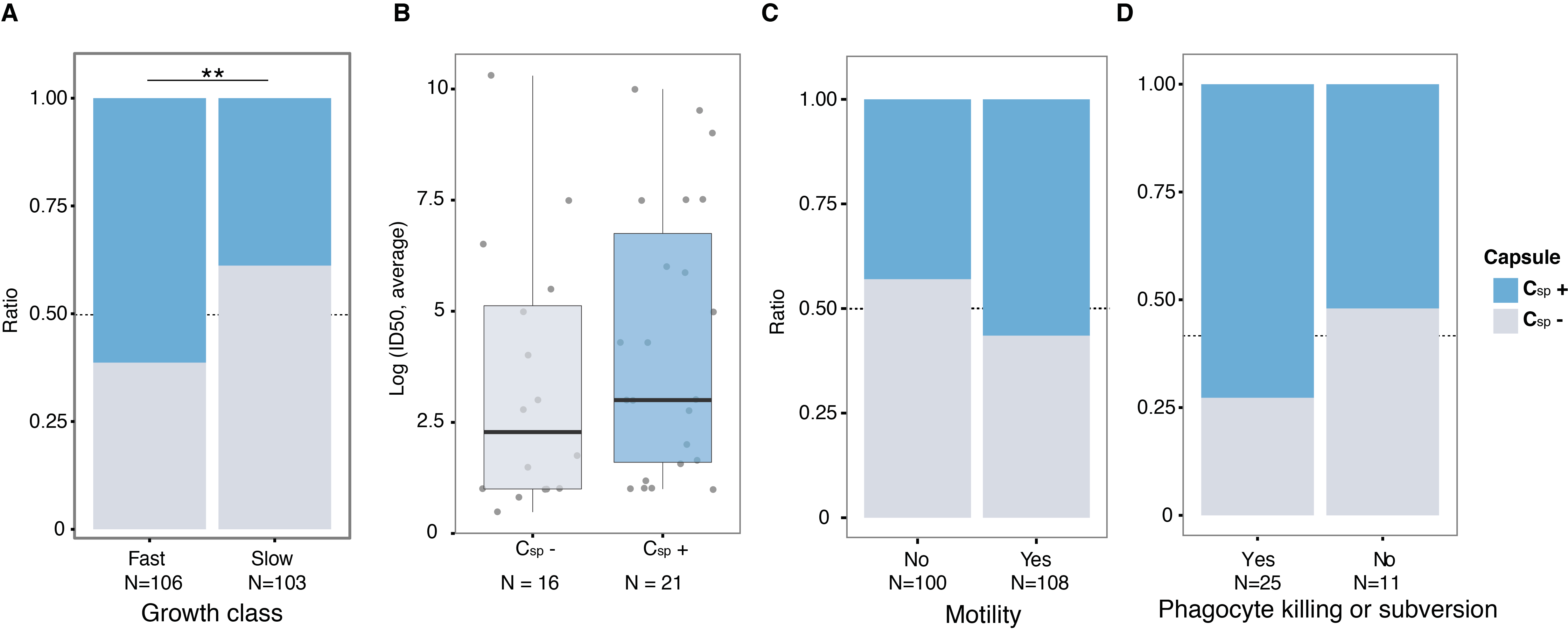

Supplement: S4 Fig — A. Frequency of Csp+ and Csp- in function of their growth class ** P < 0.01 for significant dependent evolution between growth class and presence of capsule. B. Average infection dose values (ID50) expressed in the log scale. C-D. Frequency of Csp+ and Csp- in function of the motility (C) and subversion or the ability to escape killing by phagocytes (D). Owing to lack of statistical power, association between capsules and ID50 (B) and phagocyte killing or subversion (D) were not statistically significant. ID50 data was collected for the purpose of a previous study [49]. The data was exclusively measured in human hosts, with one sole exception, the one of H. pylori. References indicating only upper or lower bounds for ID50 were discarded except when they consisted of very high lower limits or very low higher limits (e.g. <30 for H. ducreyi or >2*1010 for G. vaginalis) in which case the imprecision does not change qualitatively the character of being a very low or very high ID50 relative to the other values. ID50 values taken from immuno-compromised patients or peculiar uptakes (e.g. oral route with antacids) were excluded. To compensate for the large variance in observed values in some pathotypes, the sources of data on infectious dose were used and the average values, which are the result of arithmetic averages over the log-transformed range values, were calculated. Phagocytosis survival data, was recovered from published evidence on the ability of bacteria to survive and/or replicate in professional phagocytes and/or of being able to kill professional phagocytes. As professional phagocytes, neutrophils, monocytes, macrophages, dendritic cells, and mast cells were considered, although most evidence concerns macrophages and neutrophils. Antigenic variation or the use of specific mechanisms to actively prevent phagocytosis without killing the professional phagocyte are not included in this list. Details concerning motility was taken from the reference book [73]. (TIF) [file ppat.1006525.s015.tif]

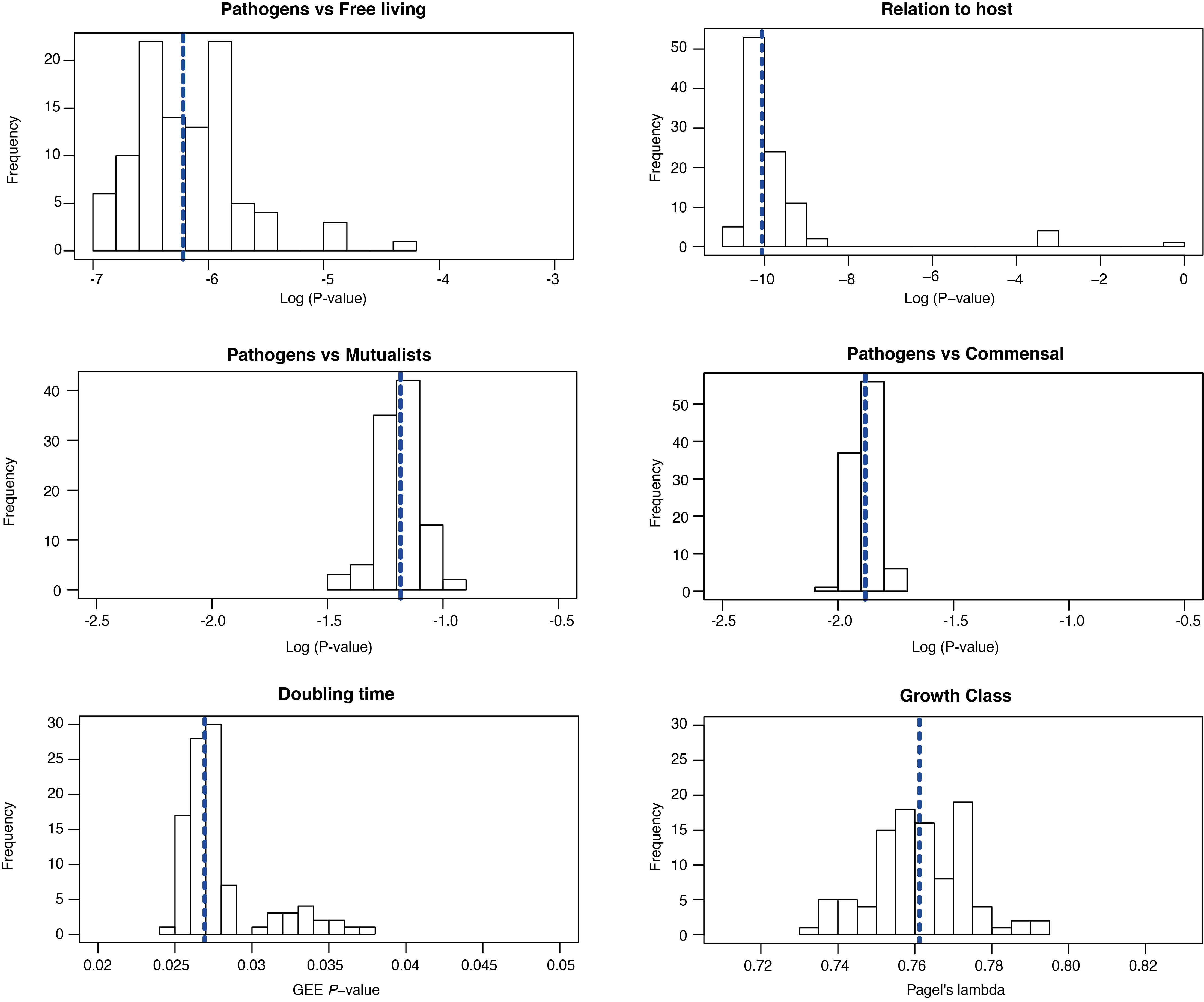

Supplement: S5 Fig — To test for the dependence between presence of capsule and bacterial lifestyle, the fitPagel function was performed on 100 trees obtained by bootstrap experiments on the multiple alignment. We plot the distribution of the corresponding P values (log-scale) in the graphs. Blue dashed lines indicate the median. To test the association of bacterial doubling time with presence of capsule, we ran compar.gee function on 100 independent trees. To analyze whether there was phylogenetic inertia in the growth class (fast or slow-growing bacteria), we ran phylosig function and Pagel’s lambda is displayed. (TIF) [file ppat.1006525.s016.tif]

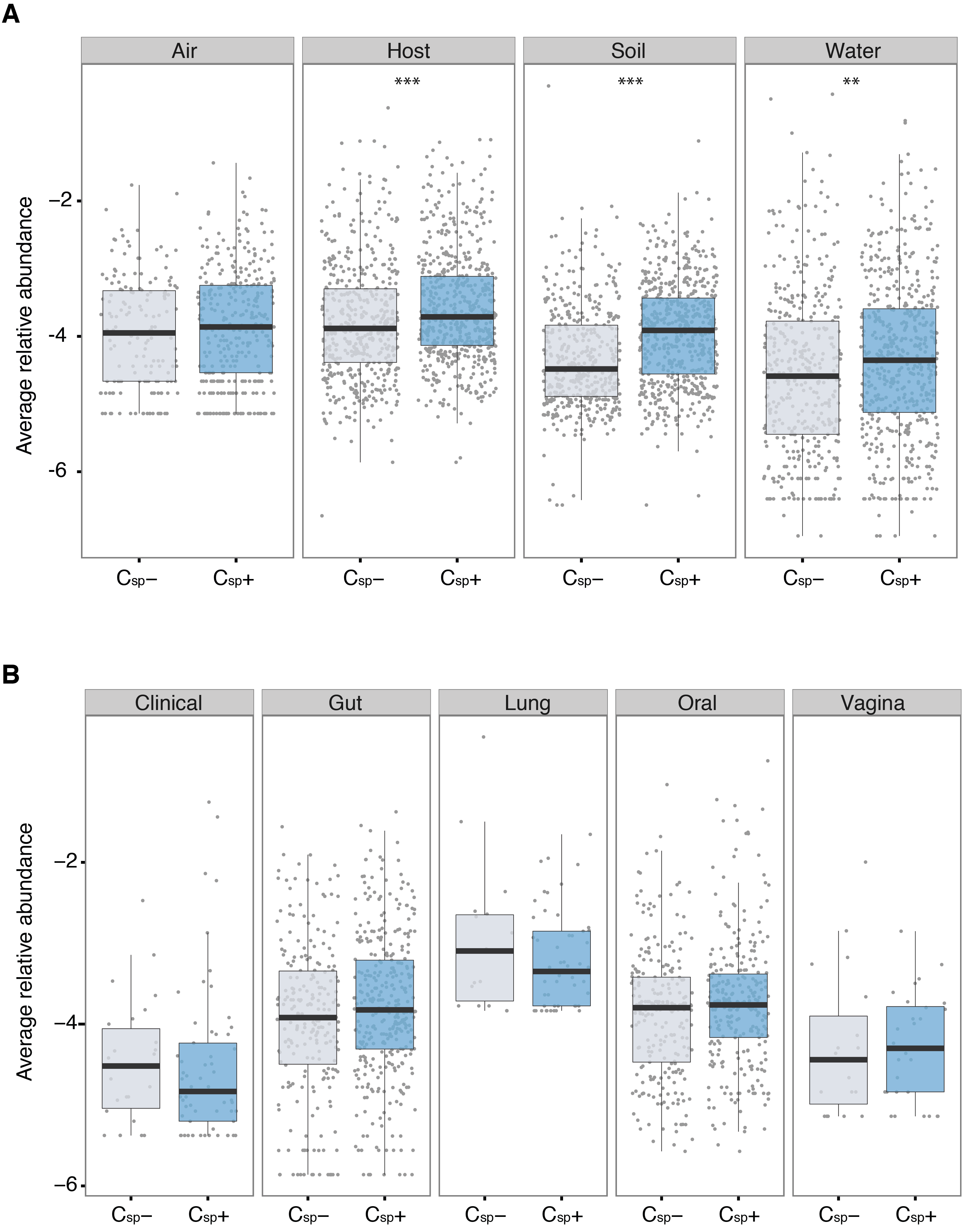

Supplement: S6 Fig — A. Relative abundance of Csp+ and Csp- across environments. Y-axis is in log scale. Statistics reflect significant differences in the relative abundance between Csp+ and Csp-, non-parametric Wilcoxon test and Benjamini & Hochberg post hoc correction ** P < 0.01, ***P < 0.0001. B. Average relative of abundance of Csp+ and Csp- across metagenomes in different body locations. (TIF) [file ppat.1006525.s017.tif]

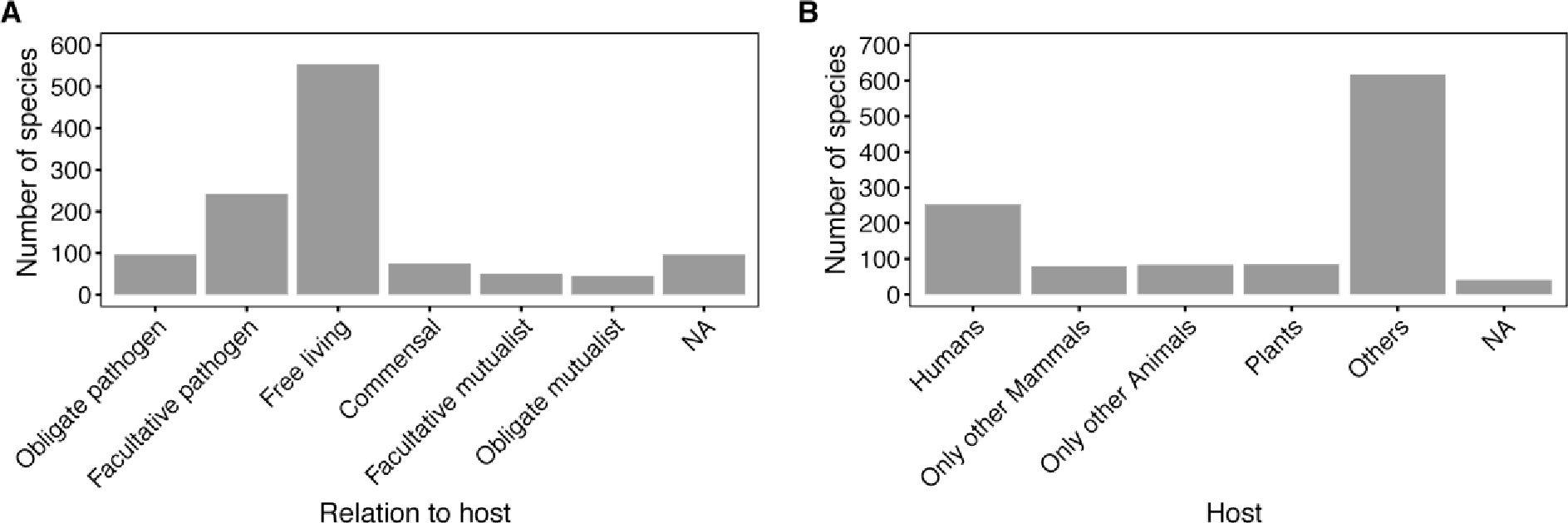

Supplement: S7 Fig — NA indicates that information was lacking or ambiguous. (TIF) [file ppat.1006525.s018.tif]
