# Supplementary material for: Abundance and co-occurrence of extracellular capsules increase environmental breadth: Implications for the emergence of pathogens
Source: PLoS Pathog. 2017 Jul 24;13(7):e1006525. doi: 10.1371/journal.ppat.1006525 (PMC5542703; doi:10.1371/journal.ppat.1006525)
Supplement: S1 Text — (PDF) [file ppat.1006525.s020.pdf]

S1 Text

Detection of false positives.

We have attempted to validate the capsule systems detected by CapsuleFinder. This was difficult due to the lack of a curated database of capsule systems and the little experimental data available in species other than a few model pathogens. We analyzed the primary scientific literature as described in the Methods section (main text). From the forty randomly chosen species in which CapsuleFinder detected a system, we only found explicit commentaries to the absence of capsule in three species. This would yield a *maximum* of ~8% of false positives. Yet, we cannot necessarily assume that these three species are *de facto* non-capsulated or that the conditions of capsular expression were not yet identified. Indeed, these three species were non-model species that may have been poorly analyzed under classical laboratory conditions, for example *Pediococcus claussenni* or *Geobacter sulforreducens*.

Additionally, we performed analysis of co-localization of capsules within a genome and observed two capsule systems of different type that occupied the same locus. The analysis of these cases revealed that they corresponded to Group I and GroupIV_e capsules. This suggests problems in the discrimination between certain capsule types that results in an increased number of capsules per genome. However, we only found seven instances of this problem out of the 2182 capsule systems detected (~0.003%), which suggests that the problem is of small importance. Further, this does not affect the classification of a genome as uncapsulated (C_{sp}-) or capsulated (C_{sp}+) but only the number of capsules per genome. This kind of false positives was not totally unexpected as different capsule types share homologous proteins involved in the polymerization machinery or exportation. For instance, both Group I and Group IV capsules rely on the polymerization activity of Wzy, or ABC capsules and Group I capsules in diderm bacteria use similar outer membrane transporters. There is no easily identifiable difference between the proteins involved in one capsule system or another.

Detection of false negatives.

Several bacterial genomes, notably those of several commensal bacteria from the gut, are known to produce at least seven different serotypes, or seven different capsules. Yet, in the direct output of CapsuleFinder, only one of these systems is detected. CapsuleFinder detects the gene clusters but these systems are not considered to be complete (i.e. they do not have the minimum mandatory genes required for a cluster to be considered as a complete system). This suggests that some mandatory elements can be shared between the different systems. The

parameters of our model could be relaxed but this leads to an increase of false positives and to the misidentification of gene clusters involved in the metabolism of sugars as polysaccharidic capsules. Hence, the unexpectedly high multiplicity of capsules of the same type in genomes pinpointed by our data may yet be under-estimated.
